# Supplementary material for: Impact of health insurance coverage for Helicobacter pylori gastritis on the trends in eradication therapy in Japan: retrospective observational study and simulation study based on real-world data
Source: BMJ Open. 2017 Jul 31;7(7):e015855. doi: 10.1136/bmjopen-2017-015855 (PMC5642792; doi:10.1136/bmjopen-2017-015855)
Supplement: Supplementary file 1 [file bmjopen-2017-015855supp001.pdf]

Supplementary appendix

Table S1. The number of primary eradication individuals, mean age, and percentage of males for each year in the JMDC and MDV databases.

		2005	2006	2007	2008	2009	2010	2011	2012	2013	2014	2015
JMDC	Number	1,001	987	1,049	1,426	2,078	3,231	3,985	4,649	19,341	21,951	21,421
	Mean age	41.5	41.1	40.4	39.2	38.4	37.7	38.4	38.3	35.8	37.1	35.9
	(SD)	(9.7)	(9.8)	(9.8)	(10.6)	(10.8)	(10.7)	(10.8)	(10.6)	(10.2)	(10.4)	(10.4)
	Male %	83	79	77	70	67	66	65	65	58	80	58
MDV	Number	-	-	-	442	2,108	5,655	7,520	9,588	36,811	52,920	55,949
	Mean age	-	-	-	58.1	57.9	59.0	59.0	59.3	60.6	60.9	61.0
	(SD)				(14.3)	(13.7)	(14.1)	(14.1)	(13.8)	(12.6)	(12.7)	(12.9)
	Male %	-	-	-	65	66	64	64	64	57	55	55

JMDC, Japan Medical Data Center; MDV, Medical Data Vision; SD, standard deviation.

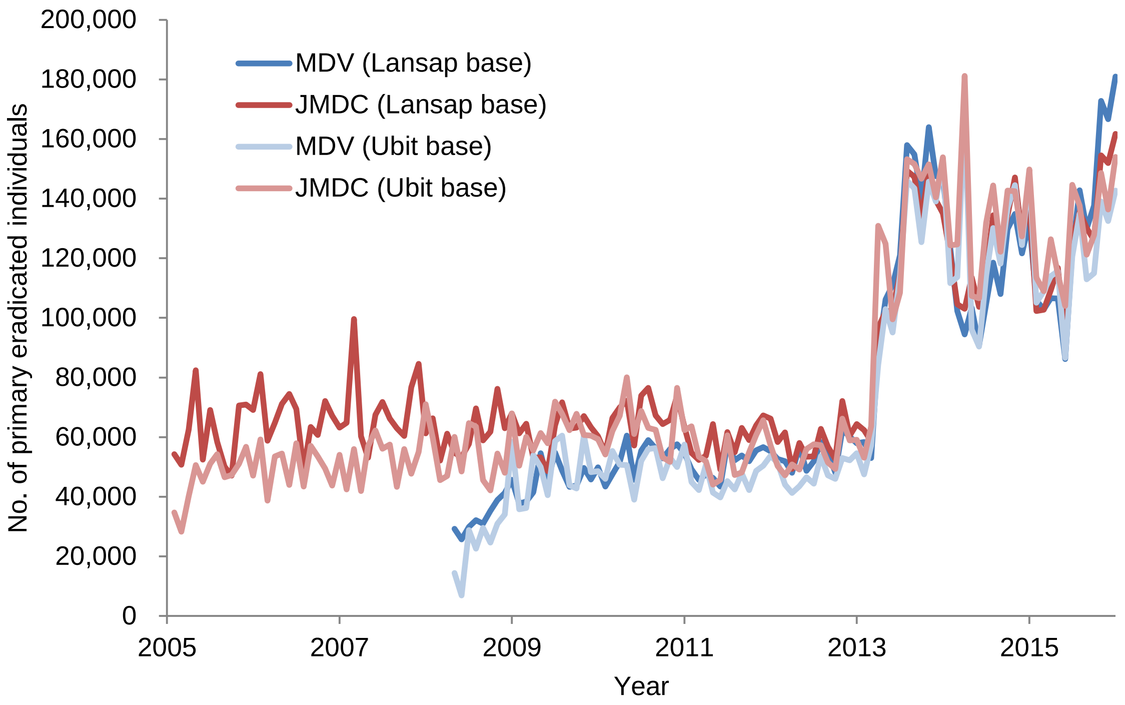

Figure S1. The number of individuals with the primary eradication of *H. pylori* according to each database.
