# Supplementary material for: Impact of health insurance coverage for Helicobacter pylori gastritis on the trends in eradication therapy in Japan: retrospective observational study and simulation study based on real-world data
Source: BMJ Open. 2017 Jul 31;7(7):e015855. doi: 10.1136/bmjopen-2017-015855 (PMC5642792; doi:10.1136/bmjopen-2017-015855)
Supplement: Author's manuscript [file bmjopen-2017-015855.draft_revisions.pdf]

BMJ Open

Impact of health insurance coverage for *Helicobacter pylori* gastritis on the trends in eradication therapy in Japan: retrospective observational study and simulation study based on real-world data.

Journal:	*BMJ Open*
Manuscript ID	bmjopen-2017-015855
Article Type:	Research
Date Submitted by the Author:	04-Jan-2017
Complete List of Authors:	Hiroi, Shinzo ; Graduate School of Medicine and Public Health, Kyoto University, Department of Pharmacoepidemiology; Takeda Pharmaceutical Company, Limited, Japan Medical Affairs<br>Sugano, Kentaro; Jichi Medical University, Department of Medicine<br>Tanaka, Shiro; Graduate School of Medicine and Public Health, Kyoto University, Department of Pharmacoepidemiology<br>Kawakami, Koji; Graduate School of Medicine and Public Health, Kyoto University, Department of Pharmacoepidemiology
**Primary Subject Heading**:	Epidemiology
Secondary Subject Heading:	Gastroenterology and hepatology
Keywords:	health insurance, <i>Helicobacter pylori</i>, <i>Helicobacter pylori</i> eradication, <i>Helicobacter pylori</i>-positive gastritis

SCHOLARONE™
Manuscripts

For peer review only

Impact of health insurance coverage for *Helicobacter pylori* gastritis on the trends in eradication therapy in Japan: retrospective observational study and simulation study based on real-world data.

Shinzo Hiroi, HEOR Head[1,3], Kentaro Sugano, Professor Emeritus[2], Shiro Tanaka, Associate Professor[1], Koji Kawakami, Professor[1]

1 Department of Pharmacoepidemiology, Graduate School of Medicine and Public Health, Kyoto University, Kyoto, Japan

2 Department of Medicine, Jichi Medical University, Tochigi, Japan

3 Japan Medical Affairs, Takeda Pharmaceutical Company, Limited, Tokyo, Japan

Corresponding author:

Koji Kawakami

Department of Pharmacoepidemiology, Graduate School of Medicine and Public Health, Kyoto University, Yoshida Konoecho, Sakyoku, Kyoto 606-8501, Japan.

Tel: +81 75 753 9469

Fax: +81 75 753 4469

E-mail address: kawakami.koji.4e@kyoto-u.ac.jp

Keywords: health insurance, *Helicobacter pylori*, *Helicobacter pylori* eradication, *Helicobacter pylori*-positive gastritis

For peer review only

Abstract

Objectives

To explore the prevalence of *Helicobacter pylori (H. pylori)* infection in Japan and the trends of its eradication therapy before and after the changes of the insurance coverage policy, first started in 2000, and expanded to cover *H. pylori*-positive gastritis in 2013. The impacts the changes brought were estimated.

Methods

Retrospective observational study and simulation study based on health insurance claims data, product sales data, and relevant studies. People who received triple therapy (amoxicillin, clarithromycin, proton-pump inhibitors or potassium-competitive acid blockers) were defined as the first-time patients for *H. pylori* eradication in two Japanese health insurance claims databases (from approximately 1.6 million and10.5 million patients). The each sales data of eradication packages and examination kits were used to estimate the number of *H. pylori*−eradicated individuals nationwide. The prevalence of *H. pylori* infection, including the future rate, was predicted using previous studies and the estimated population trend by a national institute. Cases completed prior to the policy change on insurance coverage were simulated to estimate what would have happened had there been no change in the policy.

Results

The numbers of patients first received eradication therapy were 81,119 and 170,993 from two databases. The nationwide estimated number of patients successfully eradicated was approximately 650,000 per year between 2001 and 2012, while it rapidly rose to 1,380,000 per year in 2013. The estimated prevalence of infection in 2050 is 5%; this rate was estimated to be 28% and 22% if the policy changes had not occurred in

For peer review only

2000 and 2013, respectively.

Conclusions

The impact of policy changes for *H. pylori* eradication therapy on the prevalence of infection was shown. The results suggest that insurance coverage expansion may also reduce the prevalence in other countries with a high prevalence of *H. pylori* infection if the reinfection is low.

For peer review only

Strengths and limitations of this study

- Demonstrates for the first time the impact of insurance policy expansion for *H. pylori* eradication therapy in a quantitative manner based on an analysis of nationwide real world data.
- Robust and reliable results were obtained from combinations of large-scale insurance claims databases and sales data of the most commonly used eradication treatments and test kits.
- The success rate of eradication was obtained from previous studies; therefore, the rate might be different from current clinical practice.
- The health insurance claims databases have potential biases: in one database, the information on individuals older than 65 years is limited because it is the information from employed individuals and their family members; another database included the data only from large hospitals.

For peer review only

Introduction

Throughout the world, gastric cancer is one of the most common cancers; 952,000 new patients were diagnosed in 2012.[1] The incidence of gastric cancer is higher in Asian countries; Korea, Japan, and China have the 1st, 3rd, and 5th highest rates, respectively, in the world.[2] In Japan, the prevalence and mortality of gastric cancer are constantly among the top three of all cancers. Therefore, it is considered to be one of the highest priorities in preventive policy. *Helicobacter pylori (H. pylori)* can cause gastric inflammation, which can then lead to gastric and duodenal ulcers, as well as gastric cancer.[3-5] Thus eradication of *H. pylori* is considered as an effective therapy in reducing the risk of those diseases.

Due to the concern of high gastric cancer prevalence in East Asian countries, some preventive programs have been launched to reduce the incidence of gastric cancer. In Korea, a cancer screening program was established by the government to provide for almost all people of eligible age (40 years or older for gastric cancer) with free screening or provision at minimum cost in 1999.[6] Large clinical trials and health economic studies have been conducted in China, and a consensus statement was formulated to encourage *H. pylori* eradication therapy.[6, 7] In Taiwan, the results of a community-level large screening and eradication program, as well as a health economic evaluation, support the efficacy of *H. pylori* eradication therapy.[8] In Japan, in November 2000, based on the results of diverse clinical studies,[3, 9-20] the government approved the addition of *H. pylori* eradication therapy in their insurance policy as a treatment for *H. pylori*-positive gastric ulcer and duodenal ulcer. Furthermore, insurance coverage was expanded in June 2010 to include gastric mucosa-associated lymphoid tissue lymphoma, idiopathic thrombocytopenic purpura, and post-endoscopic resection of early gastric

For peer review only

cancer, and in February 2013, to include *H. pylori*-positive gastritis based on the recommendations of Japanese guideline.[21]

Generally, health insurance reimbursement seems to have the same or greater impact on clinical practice as recommendations from diagnostic/treatment guidelines in countries where universal health insurance coverage is established, such as in Japan and Korea. Various sizes of preventative programs for gastric cancer have been implemented in the high prevalence countries for both gastric cancer and *H. pylori* infection. In some countries, *H. pylori* eradication therapy for patients with *H. pylori*-positive gastric ulcer and duodenal ulcer has been covered by national health insurance. However, eradication therapy for *H. pylori*-positive gastritis has not been covered to date in these countries other than Japan.[8] The effect of insurance coverage expansion on the prevalence of *H. pylori* infection has been evaluated in only a few studies at the community level in Japan.[22,23] Nonetheless, the national-level prevalence rate of *H. pylori* infection has not been reported and its change has not been assessed after the insurance coverage for *H. pylori* eradication therapy was expanded to include *H. pylori*-positive gastritis in 2013.The progressive insurance expansion was reported to be efficient[24] and the incidence of peptic ulcer has decreased since the change in insurance coverage policy for *H. pylori* eradication in 2000.[25] They also estimated that gastric cancer mortality would decrease based on the assumption that 50% of *H. pylori*-infected patients would receive eradication therapy.[26] However, this estimate was based on neither the observed number of patients undergoing *H. pylori* eradication nor the prevalence rate of infection. To evaluate the impact of changes to the insurance policy on the incidence of various diseases, including gastric cancer, it is necessary to elucidate the national trend of eradication therapy and the prevalence rate of infection

For peer review only

before and after the changes in the insurance policy.

The primary objective of this study was to assess how health insurance policy changes have impacted eradication therapy and the prevalence rate of *H. pylori* infection in Japan. Furthermore, the future effect, as a result of the policy changes, on the prevalence of *H. pylori* infection was evaluated. In this study, health insurance claims databases and product sales data were used to estimate the number of eradication treatments. The successful eradication rate from 2000 onwards, at which time the health insurance began its coverage for the eradication therapy, was also estimated. The prevalence rate of infection and the number of infected individuals up to 2060 were predicted as based on the above data analysis and the prevalence rate of *H. pylori* infection as reported in previous studies. Furthermore, a simulation was conducted to estimate what the probable effects would be had the policy changes not been made.

Methods

Data sources

Insurance claims databases from Japan Medical Data Center (JMDC) from January 2005 to December 2015 and Medical Data Vision (MDV) from April 2008 to December 2015 were used for the analyses. The JMDC database is a registry of health insurance claims and medical examination records for insured individuals and their families in more than 50 health insurance societies. Because this database only included information on company employees and their families, the information for those older than 65 years was limited. Also, there were no data for those older than 75 years. Until 2014, this database covered 1.6 million patients which accounted for 1% of the Japanese population. The medical database from MDV covered 10.5 million patients in 192 acute

For peer review only

care hospitals using diagnostic procedure combination/per-diem payment system (DPC/PDPS). It included 11% of acute care hospitals in Japan with the number of beds from 20 to more than 1,000. These databases included the patient's gender, age, diagnosis, prescription information, and so on. Diagnosis information is based on the International Classification of Diseases 10th Revision, and drugs are coded in the Anatomical Therapeutic Chemical Classification System. Both databases included anonymous and personally unidentifiable data.

To estimate the nationwide number of infected individuals, product sales data for the most common eradication medicine, and test kit for *H. pylori* infection were analyzed. The sales data for eradication medicine, Lansap® (Takeda Pharmaceutical Co., Ltd.), which consists of lansoprazole, amoxicillin, and clarithromycin in one package was provided by the manufacturer from December 2002 through December 2015. The data for ^{13}Urea Breath Test (UBIT®, Otsuka Pharmaceutical Co., Ltd.) was obtained as well from November 2000 through December 2015.

To determine the trend in the number of *H. pylori* infected individuals, previously published Japanese studies were used (Table 1).

Table 1. Studies on the number of *H.pylori* infected individuals and prevalence rate of infection

First Author	Title	Observation Year
Asaka M[27]	Relationship of *Helicobacter pylori* to serum pepsinogens in an asymptomatic Japanese population.	1990
Fujisawa T[28]	Changes in seroepidemiological pattern of *Helicobacter pylori* and hepatitis A virus over the last 20 years in Japan.	1974<br>1984<br>1994
Watabe H[31]	Predicting the development of gastric cancer from combining *Helicobacter pylori* antibodies and serum pepsinogen status: a prospective endoscopic cohort study.	1996
Ueda J[30]	Prevalence of *Helicobacter pylori* infection by birth year and geographic area in Japan.	2005
Shiota S[29]	*Helicobacter pylori* infection in Japan.	2009

For peer review only

Study design

This study is a retrospective observational study and simulation study based on the health insurance claims data, product sales data, and relevant published studies. The steps were as follows; firstly, the number of individuals who received the eradication therapy and those who had successful eradication were estimated based on the analyses of the health insurance claims databases and product sales data. Secondly, the trend in the number of *H. pylori* infected individuals was determined from previously published studies. Thirdly, the prevalence rate and trend of *H. pylori* infection was estimated and forecasted from the results of the first and second step. Finally, to fully evaluate the impact of the policy changes, a simulation was made considering effects which likely would have occurred without the insurance policy changes in 2000 and 2013.

Patient identification and statistical analysis

In the JMDC and MDV databases, the individuals who received triple therapy, either the primary eradication package (such as Lansap® and other packaged products), or the combination of amoxicillin, clarithromycin, and either proton-pump inhibitors or potassium-competitive acid blockers (all prescribed within the same month), were defined as individuals with primary eradication of *H. pylori*. The drugs used for the therapy were defined by product name in JMDC database and remuneration code in MDV database. The examination was defined by remuneration code, and the diagnosis for those who had eradicated was defined by name of diagnosis in both databases. To estimate the number of individuals who received primary eradication therapy in the nation, the following were calculated in these databases:

For peer review only

1) Percentage of individuals who used Lansap® for primary eradication therapy to all individuals who received the primary eradication.

2) Percentage of individuals who received UBIT® for the *H. pylori* test after eradication to all individuals who took the *H. pylori* test.

To calculate the number of individuals who achieved successful eradication, the following were assumed:

- The primary and secondary success rates of eradication for this study were presumed to be 75% and 90%, respectively, based on previous studies in Japan.[25, 27] Secondary eradication was premised to be performed for all those who failed primary eradication. Therefore, the success rate of the eradications was estimated to be 98% of the primary eradication, and the percentage was used as the success rate in this study.
- New infection in adulthood was reported to be rare[32] and reinfection per year after the eradication therapy in Japan is reported to be approximately 1%;[33,34] however it was assumed to be 0% in this study.

The nationwide number of individuals who received primary eradication was estimated based on 1) and 2) with sales data of Lansap® and UBIT® as follows:

- The monthly number of individuals who received primary eradication from January 2010 was calculated as the mean of four estimates (Lansap®-base from MDV and JMDV, UBIT®-base from MDV and JMDC).
- The monthly number of individuals who received primary eradication from January 2006 to December 2009 was calculated as the mean of two estimates from the JMDC database (Lansap®-base and UBIT®-base).
- The monthly number of individuals who received primary eradication from

For peer review only

November 2000 to December 2005 was extrapolated using the sales number of UBIT® in each month and the UBIT® share rate in 2006 on the assumption that the share rate in this period was the same as that in 2006.

The formula used is as follows:

$$(\text{Successful Eradication Number})_{YM} = (\text{Primary Successful Eradication Number})_{YM} + (\text{Secondary Successful Eradication Number})_{YM}$$

$$(\text{Primary Successful Eradication Number})_{YM} = (\text{Primary Eradication Number})_{YM} \times (\text{Primary Eradication Success Rate, 75\%})$$

$$(\text{Secondary Successful Eradication Number})_{YM} = [(\text{Primary Eradication Number})_{YM} - (\text{Primary Successful Eradication Number})_{YM}] \times (\text{Secondary Eradication Success Rate, 90\%})$$

$$(\text{Primary Eradication Number})_{YM} = [(\text{Primary Eradication Number with Lansap®})_{YM,JMDC} + (\text{Primary Eradication Number with Lansap®})_{YM,MDV} + (\text{Primary Eradication Number with UBIT®})_{YM,JMDC} + (\text{Primary Eradication Number with UBIT®})_{YM,MDV}] / 4$$

$$(\text{Primary Eradication Number with Lansap®})_{YM,Database} = (\text{Sales Number of Lansap®})_{YM} / (\text{Share of Lansap®})_{YM,Database}$$

$$(\text{Primary Eradication Number with UBIT®})_{YM,Database} = (\text{Examination Number})_{YM,Database} \times (\text{Ratio of Primary Eradication to Total Examination})_{YM,Database}$$

$$(\text{Examination Number})_{YM,Database} = (\text{Sales Number of UBIT®})_{YM} / (\text{Share of UBIT®})_{YM,Database}$$

where YM is year month, and Database = JMDC or MDV database.

The number of infected individuals and prevalence rate of infection were estimated based on the previous Japanese studies shown in Table 1. The number of

For peer review only

infected individuals until March 2013 was estimated from previous studies[27 -31] and vital statistics in Japan conducted by Ministry of Health, Labour and Welfare.[35] An exponential decay approximation curve was calculated based on the results of the previous studies until 2000. After 2000, the estimated mean monthly number of individuals who achieved successful eradication from January 2001 to March 2013 was taken into account. The number of infected individuals from April 2013 was estimated using the estimated mean monthly number of individuals who achieved successful eradication from April 2013 to December 2015, giving consideration to the decrease in the number of infected individuals due to death. It was calculated as follows:

$$(\text{Infection Number})_{\text{CY}} = \frac{\sum_{\text{DOBYR}} \text{National Population}_{\text{DOBYR, CY}} \times \text{Prevalence Rate}_{\text{DOBYR, CY}}}{\sum_{\text{DOBYR}} \text{National Population}_{\text{DOBYR, CY}}}$$

where $(\text{Prevalence Rate})_{\text{DOBYR,CY}}$ is the prevalence rate of *H.pylori* infection by birth year in each observation year calculated in this study, and $(\text{National Population})_{\text{DOBYR,CY}}$ is the population by birth year in each observation year; where DOBYR is birth year by 5 years, and CY is the calendar year of observation of each study. An exponential parameter that minimizes the sum of squared distances from $(\text{Infection Number})_{\text{CY}}$ was calculated (least squares method), assuming the decrease of the number of *H. pylori* infected individuals by any reason other than eradication (*i.e.,* aging) followed an exponential function and the decrease of that by eradication was constant from 2001 through 2013.

Assuming that *H. pylori* infection would be decreasing exponentially after 2015 (by consideration of the natural decrease due to the death of older infected individuals), a simulation was performed using the number of infected individuals obtained in the previous section and the population forecast from the National Institute of Population

For peer review only

and Social Security Research[36] to predict the number of infected individuals in the future. The prevalence rate of infection was also simulated for the case of no policy change regarding insurance coverage in 2000 and 2013. This was simulated as follows:

1) The number of *H. pylori* infected individuals calculated in the previous section was taken to be $(\text{Infection Number})_{CY}$ before 2013.
2) The number of *H. pylori* infected individuals from 2013 through 2015 was assumed to be decreased from $(\text{Infection Number})_{CY}$ before 2013, based on the estimated number of individuals who achieved successful eradication by the analysis of the JMDC and MDV databases and sales data of drugs.
3) If Case 0, $(\text{Prevalence Rate})_{CY,case} = (\text{Infection Number})_{CY} / (\text{National Population})_{CY}$, in $1975 \leq CY \leq 2000$; or $(\text{Prevalence Rate})_{CY,case} / (\text{Prevalence Rate})_{CY-1,case} = (\text{Infection Number})_{CY-1} / (\text{Infection Number})_{CY-2}$, in $2016 \leq CY$.
4) If Case 1, $(\text{Prevalence Rate})_{CY,case} = (\text{Infection Number})_{CY} / (\text{National Population})_{CY}$, in $1975 \leq CY \leq 2000$;or $(\text{Prevalence Rate})_{CY,case} / (\text{Prevalence Rate})_{CY-1,case} = (\text{Infection Number})_{CY-1} / (\text{Infection Number})_{CY-2}$ in $2001 \leq CY$.
5) If Case 2, $(\text{Prevalence Rate})_{CY,case} = (\text{Infection Number})_{CY} / (\text{National Population})_{CY}$, in $1975 \leq CY \leq 2012$;or $(\text{Prevalence Rate})_{CY,case} / (\text{Prevalence Rate})_{CY-1,case} = (\text{Infection Number})_{CY-1} / (\text{Infection Number})_{CY-2}$ in $2013 \leq CY$.

Case 0 is with the current policy; Case 1 or 2 represent the case in which policy change had not occurred in 2000 or 2013.

Statistical analysis was carried out using Excel 2010 (Microsoft, Redmond, WA, USA) and SAS version 9.2 (SAS institute, Cary, NC, USA).

Results

For peer review only

Patient characteristics

The total number of individuals who received primary eradication was 81,119 (mean age 36.8 years; males 61%) from the JMDC database, and 170,993 (mean age 60.6 years; males 57%) from the MDV database. The characteristics for each year are shown in Table S1.

Trend in the number of individuals with the primary eradication therapy and successful eradication

The difference among the four (Lansap®-base from MDV and JMDV, UBIT®-base from MDV and JMDC) estimated numbers of individuals who received primary eradication was confirmed to be minimal after 2010 (Figure S1). The nationwide number of individuals who had successful eradication (both first-line and second-line) was estimated as shown in Figure 1. The number was approximately 650,000 per year between 2001 and 2012, which has reached a steady state of approximately 700,000 per year after an increase in 2006. However, there was a slight decrease in 2011. It markedly increased to 1,380,000 in 2013, which is more than double the number observed in 2012. In the diagnoses for those who had successful eradication treatment (Figure 1), gastritis accounted for more than half of the diagnoses since 2013. The average number of individuals who received successful eradication treatment up to March 2013 was 54,000 per month, while it was 124,000 per month after March 2013. The cumulative total of individuals who received successful eradication treatment was more than 10 million up to September 2014.

Trend in the number of infected individuals and the prevalence rate of infection

For peer review only

Figure 2a illustrates the prevalence rate of *H. pylori* infection by birth year from previous studies. This shows higher prevalence rates of infection in the cohorts with earlier birth years. Also, there was a tendency for the difference of prevalence rates of infection among studies to be larger in those with an earlier birth year, and the rate was lower in later observations. The overall estimated prevalence rate of infection was lower in later years (Figure 2b). The lines were fitted after taking the effect of insurance policy change in November 2011 into account, shown in Figure 2b.

Trend and prediction of H. pylori infection and the effect of insurance policy changes

The pattern of the number of infected individuals from 2016 in Japan was predicted (blue broken line in Figure 3) based on the trend in the number of infected individuals derived from the results above and the population forecast[35]. The patterns, in the case without policy changes in insurance coverage in 2000 and 2013, were also simulated (red and green broken lines in Figure 3). The simulation showed that the number of infected individuals would decrease to 16,200,000 individuals in 2030, or 14% of the population, and further decrease to 5% of the population in 2050. These figures would have been 28% and 21% in 2030 if the policy changes had not occurred in 2000 and 2013, respectively.

Discussion

This study described the status of eradication therapy and trend of *H. pylori* infection using large insurance claims databases that reflected actual clinical practice at the national level in Japan. The analysis showed that the prevalence rate of *H. pylori* infection has decreased after the approval to include eradication therapy in the insurance

For peer review only

policy in 2000. The number of successful eradications more than doubled immediately after insurance coverage for *H. pylori* eradication therapy was expanded in 2013. The simulation indicated that the prevalence rate of *H. pylori* infection would decrease and reach approximately 14% in 2030 and 5.4% in 2050.

Although it is difficult to compare the prevalence rate of infection among studies due to time and sample difference, it is worthwhile to compare the rate with other countries. The prevalence rate of *H. pylori* infection varies markedly in different countries; in general, it is higher in developing countries and lower in developed countries.[37] The prevalence rate of infection was reported to be 92% in Bangladesh,[38] 75% in Vietnam,[39] 58% in China,[40] and 60% in Korea,[41] while it was 15% in Australia[42] and 8% in the USA.[43] Although the estimate of the prevalence rate of *H. pylori* infection in our study was 43% in Japan in 2000, based on our simulation, the prevalence rate of infection in Japan in 2030 with expanded insurance coverage would be almost the same as the Australian rate (15%), and it would reach the North American level (8%) by approximately 2050.

The estimate from the previous studies indicated a higher prevalence rate of infection in older cohorts, which can be explained by environmental factors such as poor sanitation.[44,45] It has also been suggested that nowadays *H. pylori* infection occurs in childhood in Japan.[46,47] As a result, the number of infections would have naturally decreased even without eradication therapy due to the death of older infected individuals. However, our simulation showed that the prevalence rate of infection would have been higher than 14% and 7% in 2030 if the policy changes had not occurred in 2000 and 2013 respectively. It was therefore evident that those insurance policy changes had contributed to the reduction in the prevalence of *H. pylori* infection. Japan has

For peer review only

established a universal health insurance coverage system, which means that by law all Japanese residents are entitled to health insurance coverage for medical treatments. This system has a great impact on the dissemination of medical treatments; consequently the use of eradication therapy is highly influenced by the presence of the health insurance and coverage for diseases.

The results of this study could be a good indicator for the implementation of insurance coverage for eradication of *H. pylori* in countries where *H. pylori* is prevalent, especially the East Asian countries. Countries such as Korea, China, and Taiwan, have been conducting clinical trials of *H. pylori* eradication; however, they have not yet established any policy for *H. pylori* eradication. This study is likely to be used as one of the references in considering effect on policy change in such countries. This study might also be important in providing direction for future research in Japan. In 2016, the revised edition of the Japanese Guideline for Diagnosis and Treatment for *H. pylori* infection was published following that of 2009. In the latest guideline, the expansion of insurance coverage for the treatment for *H. pylori* gastritis in 2013 is described.[48] Also, a regimen with potassium-competitive acid blocker (P-CAB)-based triple therapy, which is newly described in the guideline, demonstrated a high eradication rate compared to the conventional proton pump inhibitor-based triple therapy.[48,49] It seems that the prevalence rate of infection could be further reduced in Japan.

Comparison with other studies

There have been some community level studies investigating the status of eradication in clinical practices in Japan.[22, 23] Nevertheless, to our knowledge, there was no study describing the status of eradication therapy at the national level. Asaka *et al.* have

For peer review only

reported that the insurance policy change could increase the number of eradications, reduce the number of infected individuals, and decrease mortality from gastric cancer.[24,25] They estimated that the number of deaths from gastric cancer would reach 60,000 in 2020 without any countermeasures, while it would be half if 50% of infected individuals receive eradication therapy. However, this assumption was not based on actual observations. Our study, using real world data reflecting actual medical practice, showed a rapid increase in patients receiving *H. pylori* eradication, after the insurance policy change in 2013.

Strengths and limitations of this study

Here, a national level evaluation using real world data, allowed us to analyze the impact of insurance policy expansion for *H. pylori* eradication therapy in a quantitative manner. Furthermore, robust and reliable results were obtained from combinations of large-scale insurance claims databases and sales data of the most commonly used eradication treatments and test kits.

However, this study has several limitations. First, the success rate of eradication was obtained from previous studies;[25,27] thus, the rate might be different from clinical practice. Nevertheless, the success rate in this study is believed to be close to the actual rate. Second, the health insurance claims databases have potential biases. The information available was limited for those older than 65 years in the JMDC database because it consisted of information on employed individuals and their family members. In addition, those who were self-employed and employees in small-to-medium-sized enterprises were not included. The information in the MDV database was obtained from the hospital using DPC/PDPS. Indeed, the mean ages of

For peer review only

individuals who received primary eradication in both databases were different. However, the product share rates calculated in both databases were very similar after 2010, and the estimates of the number of individuals with the primary eradication were almost identical in both databases. Therefore, the impact of the different age distribution was believed to be minimal, and the estimates are believed to be accurate.

Despite these limitations, this study used the information from reliable clinical studies and large databases covering a significant number of Japanese citizens. Consequently, it is believed that the information presented here reflects the clinical status in Japan.

Conclusion and policy implications

This study described and forecasted the trend of *H. pylori* eradication therapy and assessed the impact of insurance policy change on the prevalence rate of *H. pylori* infection. It has demonstrated that the policy change was associated with a reduction in the prevalence rate of *H. pylori* infection in Japan. Furthermore it is expected to lead to a reduction in the incidence of gastric cancer, so the data in this study may be cited in future studies such as a prediction of gastric cancer mortality. We believe that nation-wide health insurance coverage for *H. pylori* eradication could reduce the prevalence rate of *H. pylori* infection and possibly lead to a reduction in the incidence of gastric cancer in other countries as well.

For peer review only

Acknowledgements

The authors thank Takeda's collaborator, Otsuka Pharmaceutical Co., Ltd. for providing sales data of urea breath test diagnostic kits (UBIT®). The authors thank Kosuke Iwasaki and Tomomi Takeshima, employees of Milliman Inc., for analyzing the database, and writing and editorial suppport, respectively, funded by Takeda Pharmaceutical Co., Ltd.

Contributors

SH, KS and KK contributed to the concept and design of the study.

SH contributed to acquisition of data.

SH contributed to the analysis.

SH, KS, and ST contributed to interpretation of data.

SH, KS, ST and KK contributed to the writing of the manuscript and critical revision of the manuscript.

All authors approved the final version of the manuscript.

KK is the guarantor of the article.

Funding

This study was sponsored by Takeda Pharmaceutical Co., Ltd.

Competing interests

SH is an employee of Takeda Pharmaceutical Co., Ltd.

KS has received research grants from Eisai, Daiichi Sankyo Pharm, and Takeda Pharmaceutical Co., Ltd. He has also received lecture fees from Astellas Pharma,

For peer review only

Fujifilm, and Takeda Pharmaceutical Co., Ltd.

ST has no personal interests to declare.

KK received research funds from Dainippon Sumitomo Pharma, Olympus, Stella Pharma, Medical Platform Co., Novartis Pharmaceutical K.K., Bayer, and Maruho; honorarium from Astellas, Daiichi Sankyo Pharm, Taisho Pharmaceutical, Eisai, Novartis Pharmaceutical K.K., Mitsubishi Tanabe Pharma, Takeda Pharmaceutical Co., Ltd, Sanofi K.K.; consult fees from Olympus, Kyowa Hakko Kirin, Kaken Pharmaceutical, and Otsuka Pharmaceutical Co., Ltd.

There are no patents, products in development or marketed products to declare, relevant to those companies.

Ethical approval

This study was approved by Ethic Committee, Kyoto University Graduate School and Faculty of Medicine (R0126-1). This study was exempted from obtaining individual informed consent based on Ethical Guidelines for Medical and Health Research Involving Human Subjects by Ministry of Education, Culture, Sports, Science and Technology, and Ministry of Health, Labour, and Welfare.

Provenance and peer review

Not commissioned; externally peer reviewed.

Data sharing statement

No additional data are available.

For peer review only

Transparency

The lead author (KK) affirms that the manuscript is an honest, accurate, and transparent account of the study reported; that no important aspects of the study have been omitted; and that any discrepancies from the study as planned have been explained.

This is an Open Access article distributed in accordance with the Creative Commons Attribution Non Commercial (CC BY-NC 3.0) license, which permits others to distribute, remix, adapt, build upon this work non-commercially, and license their derivative works on different terms, provided the original work is properly cited and the use is non-commercial. See: http://creativecommons.org/licenses/by-nc/3.0/.

For peer review only

References

1. Ferlay J, Soerjomataram I, Dikshit R, et al. Cancer incidence and mortality worldwide: sources, methods and major patterns in GLOBOCAN 2012. *Int J Cancer* 2015;136:E359-86 doi: 10.1002/ijc.29210.

2. World Cancer Research Fund International. Stomach cancer statistics. 2016. http://www.wcrf.org/int/cancer-facts-figures/data-specific-cancers/stomach-cancer-statistics.

3. Fukase K, Kato M, Kikuchi S, et al. Effect of eradication of *Helicobacter pylori* on incidence of metachronous gastric carcinoma after endoscopic resection of early gastric cancer: an open-label, randomised controlled trial. *Lancet* 2008;372:392-97 doi: 10.1016/S0140-6736(08)61159-9.

4. Marshall BJ, Armstrong JA, McGechie DB, et al. Attempt to fulfil Koch's postulates for pyloric *Campylobacter*. *Med J Aust* 1985;142:436-39.

5. Morris A, Nicholson G. Ingestion of *Campylobacter pyloridis* causes gastritis and raised fasting gastric pH. *Am J Gastroenterol* 1987;82:192-99.

6. IARC Working Group. *Helicobacter pylori* eradication as a strategy for preventing gastric cancer. 2014. https://www.iarc.fr/en/publications/pdfs-online/wrk/wrk8/Helicobacter_pylori_Eradication.pdf.

7. Liu WZ, Xie Y, Cheng H, et al. Fourth Chinese National Consensus Report on the management of *Helicobacter pylori* infection. *J Dig Dis* 2013;14:211-21 doi: 10.1111/1751-2980.12034.

8. Lee S-Y. Current progress toward eradicating *Helicobacter pylori* in East Asian countries: differences in the 2013 revised guidelines between China, Japan, and

For peer review only

South Korea. *World J Gastroenterol* 2014;20:1493-502 doi: 10.3748/wjg.v20.i6.1493.

9. Asaka M, Kato M, Sugiyama T, et al. Follow-up survey of a large-scale multicenter, double-blind study of triple therapy with lansoprazole, amoxicillin, and clarithromycin for eradication of *Helicobacter pylori* in Japanese peptic ulcer patients. *J Gastroenterol* 2003;38:339-47 doi: 10.1007/s005350300061.

10. Emilia G, Longo G, Luppi M, et al. *Helicobacter pylori* eradication can induce platelet recovery in idiopathic thrombocytopenic purpura. *Blood* 2001;97:812-14 doi: 10.1182/blood.V97.3.812.

11. Gasbarrini A, Franceschi F, Tartaglione R, et al. Regression of autoimmune thrombocytopenia after eradication of *Helicobacter pylori*. *Lancet* 1998;352:878-78 doi: 10.1016/S0140-6736(05)60004-9.

12. Hentschel E, Brandstatter G, Dragosics B, et al. Effect of ranitidine and amoxicillin plus metronidazole on the eradication of *Helicobacter pylori* and the recurrence of duodenal ulcer. *N Engl J Med* 1993;328:308-12 doi: 10.1056/NEJM199302043280503.

13. Leodolter A, Kulig M, Brasch H, et al. A meta‐analysis comparing eradication, healing and relapse rates in patients with *Helicobacter pylori*‐associated gastric or duodenal ulcer. *Aliment Pharmacol Ther* 2001;15:1949-58 doi: 10.1046/j.1365-2036.2001.01109.x.

14. Malfertheiner P, Megraud F, O'Morain C, et al. Current concepts in the management of *Helicobacter pylori* infection: the Maastricht III Consensus Report. *Gut* 2007;56:772-81 doi: 10.1136/gut.2006.101634.

15. Miwa H, Sakaki N, Sugano K, et al. Recurrent peptic ulcers in patients following

For peer review only

successful *Helicobacter pylori* eradication: a multicenter study of 4940 patients. *Helicobacter* 2004;9:9-16 doi: 10.1111/j.1083-4389.2004.00194.x.

16. Ogura K, Hirata Y, Yanai A, et al. The effect of *Helicobacter pylori* eradication on reducing the incidence of gastric cancer. *J Clin Gastroenterol* 2008;42:279-83 doi: 10.1097/01.mcg.0000248006.80699.7f.
17. Take S, Mizuno M, Ishiki K, et al. The effect of eradicating *Helicobacter pylori* on the development of gastric cancer in patients with peptic ulcer disease. *Am J Gastroenterol* 2005;100:1037-42 doi: 10.1111/j.1572-0241.2005.41384.x.
18. Takenaka R, Okada H, Kato J, et al. *Helicobacter pylori* eradication reduced the incidence of gastric cancer, especially of the intestinal type. *Aliment Pharmacol Ther* 2007;25:805-12 doi: 10.1111/j.1365-2036.2007.03268.x.
19. Wong BC-Y, Lam SK, Wong WM, et al. *Helicobacter pylori* eradication to prevent gastric cancer in a high-risk region of China: a randomized controlled trial. *JAMA* 2004;291:187-94 doi: 10.1001/jama.291.2.187.
20. Wotherspoon AC, Diss TC, Pan L, et al. Regression of primary low-grade B-cell gastric lymphoma of mucosa-associated lymphoid tissue type after eradication of *Helicobacter pylori*. *Lancet* 1993;342:575-77 doi: 10.1016/0140-6736(93)91409-F.
21. Asaka M, Kato M, Takahashi S, et al. Guidelines for the management of *Helicobacter pylori* infection in Japan: 2009 revised edition. *Helicobacter.* 2010;15:1-20 doi: 10.1111/j.1523-5378.2009.00738.x.
22. Hanamure Y. [Eradication therapy in the era of *Helicobacter pylori* infection -Its issues-]. *Helicobacter Research* 2014;18. Japanese.
23. Fujimori K. [The changes in *Helicobacter pylori* infection treatment: Is the number of infection reduced?]. *Helicobacter Research* 2004;18. Japanese.

For peer review only

24. Asaka M, Kato M, Sakamoto N. Roadmap to eliminate gastric cancer with *Helicobacter pylori* eradication and consecutive surveillance in Japan. *J Gastroenterol* 2014;49:1-8 doi: 10.1007/s00535-013-0897-8.

25. Sugano K, Osawa H, Satoh K. Clinical Management of *Helicobacter pylori*-The Japanese Perspective. *Dig Dis* 2014;32:281-89 doi: 10.1159/000357859.

26. Asaka M, Mabe K, Matsushima R, et al. *Helicobacter pylori* eradication to eliminate gastric cancer: the Japanese strategy. *Gastroenterol Clin North Am* 2015;44:639-48 doi: 10.1016/j.gtc.2015.05.010.

27. Asaka M, Kimura T, Kudo M, et al. Relationship of *Helicobacter pylori* to serum pepsinogens in an asymptomatic Japanese population. *Gastroenterology* 1992;102:760-66 doi: 10.1016/0016-5085(92)90156-S.

28. Fujisawa T, Kumagai T, Akamatsu T, et al. Changes in seroepidemiological pattern of *Helicobacter pylori* and hepatitis A virus over the last 20 years in Japan. Am J Gastroenterol 1999;94:2094-99 doi: 10.1111/j.1572-0241.1999.01283.x.

29. Shiota S, Murakawi K, Suzuki R, et al. *Helicobacter pylori* infection in Japan. Expert Rev Gastroenterol Hepatol 2013;7:35-40 doi: 10.1586/egh.12.67.

30. Ueda J, Gosho M, Inui Y, et al. Prevalence of *Helicobacter pylori* infection by birth year and geographic area in Japan. Helicobacter 2014;19:105-10 doi: 10.1111/hel.12110.

31. Watabe H, Mitsushima T, Yamaji Y, et al. Predicting the development of gastric cancer from combining *Helicobacter pylori* antibodies and serum pepsinogen status: a prospective endoscopic cohort study. Gut 2005;54:764-68 doi: 10.1136/gut.2004.055400.

32. Cave DR. How is *Helicobacter pylori* transmitted? *Gastroenterology*

For peer review only

1997;113:S9-14 doi: 10.1016/S0016-5085(97)80004-2.

33. Take S, Mizuno M, Ishiki K, et al. Reinfection rate of *Helicobacter pylori* after eradication treatment: a long-term prospective study in Japan. *J Gastroenterol* 2012;47:641-6 doi: 10.1007/s00535-012-0536-9.

34. Okimoto T, Murakami K, Sato R, et al. Is the recurrence of *Helicobacter pylori* infection after eradication therapy resultant from recrudescence or reinfection, in Japan. *Helicobacter* 2003;8:186-91 doi: 10.1046/j.1523-5378.2003.00143.x.

35. Ministry of Health, Labour and Welfare. [Summary Report of Vital Statistics of Japan 2013 (final data)]. 2014. http://www.mhlw.go.jp/toukei/saikin/hw/jinkou/kakutei13/index.html. Japanese.

36. National Institute of Population and Social Security Research. [Future Population Prediction in Japan]. 2012. http://www.ipss.go.jp/syoushika/tohkei/newest04/sh2401top.html. Japanese.

37. Fock KM, Ang TL. Epidemiology of *Helicobacter pylori* infection and gastric cancer in Asia. *J Gastroenterol Hepatol* 2010;25:479-86 doi: 10.1111/j.1440-1746.2009.06188.x.

38. Ahmad MM, Rahman M, Rumi AK, et al. Prevalence of *Helicobacter pylori* in asymptomatic population--a pilot serological study in Bangladesh. *J Epidemiol* 1997;7:251-54 doi: 10.2188/jea.7.251.

39. Hoang TTH, Bengtsson C, Phung DC, et al. Seroprevalence of *Helicobacter pylori* infection in urban and rural Vietnam. *Clin Diagn Lab Immunol* 2005;12:81-85 doi: 10.1128/CDLI.12.1.81-85.2005.

40. Wang K-j, Wang R-t. Meta-analysis on the epidemiology of *Helicobacter pylori* infection in China. *Zhonghua Liu Xing Bing Xue Za Zhi* 2003;24:443-46 doi:

For peer review only

10.1111/j.1440-1746.2010.06608.x.

41. Yim JY, Kim N, Choi SH, et al. Seroprevalence of *Helicobacter pylori* in South Korea. *Helicobacter* 2007;12:333-40 doi: 10.1111/j.1523-5378.2007.00504.x.

42. Moujaber T, MacIntyre CR, Backhouse J, et al. The seroepidemiology of *Helicobacter pylori* infection in Australia. *Int J Infect Dis* 2008;12:500-04 doi: 10.1016/j.ijid.2008.01.011.

43. Sonnenberg A, Lash RH, Genta RM. A national study of *Helicobactor pylori* infection in gastric biopsy specimens. *Gastroenterology* 2010;139:1894-901 doi: 10.1053/j.gastro.2010.08.018.

44. Goh KL, Chan WK, Shiota S, et al. Epidemiology of *Helicobacter pylori* infection and public health implications. *Helicobacter* 2011;16:1-9 doi: 10.1111/j.1523-5378.2011.00874.x.

45. Ueda M, Kikuchi S, Kasugai T, et al. *Helicobacter pylori* risk associated with childhood home environment. *Cancer Sc* 2003;94:914-8. doi: 10.1111/j.1349-7006.2003.tb01375.x

46. Konno M, Fujii N, Yokota S, et al. Five-year follow-up study of mother-to-child transmission of *Helicobacter pylori* infection detected by a random amplified polymorphic DNA fingerprinting method. *J Clin Microbiol* 2005;43:2246-50. doi: 10.1128/JCM.43.5.2246-2250.2005

47. Okuda M1, Miyashiro E, Booka M, et al. *Helicobacter pylori* colonization in the first 3 years of life in Japanese children. *Helicobacter* 2007;12:324-7. doi: 10.1111/j.1523-5378.2007.00510.x

48. The committee of the Japanese Society for Helicobacter Research. [Guidelines for diagnosis and treatment of *H. pylori* infection in Japan: 2016 revised edition]. Tokyo:

For peer review only

Sentan Igaku-Sha; 2016. Japanese.

49. Murakami K, Sakurai Y, Shiino M, et al. Vonoprazan, a novel potassium-competitive acid blocker, as a component of first-line and second-line triple therapy for *Helicobacter pylori* eradication: a phase III, randomised, double-blind study. *Gut* 2016;65:1439-46 doi: 10.1136/gutjnl-2015-311304.

For peer review only

Figure legends

Figure 1. Annual number of individuals who had successful eradication (both first-line and second-line). *Others: gastric mucosa associated lymphoid tissue lymphoma, idiopathic thrombocytopenic purpura, and post-endoscopic resection of early gastric cancer.

Figure 2. Trends in (a) the percentages of *H. pylori* infection by birth year based on previous studies and (b) the nationwide number of infected individuals as estimated based on the previous studies. *Data from the study by Watabe et al.[31] were excluded in (a) as that study divided the age group into 2 age groups: below and above age 60. *H. pylori, Helicobacter pylori.*

Figure 3. Trend and prediction of the number of *H. pylori* infected individuals. *Case 0, in current policy; †case 1, if policy change had not occurred in 2000; ‡case 2, if policy change had not occurred in 2013. *H. pylori, Helicobacter pylori.*

For peer review only

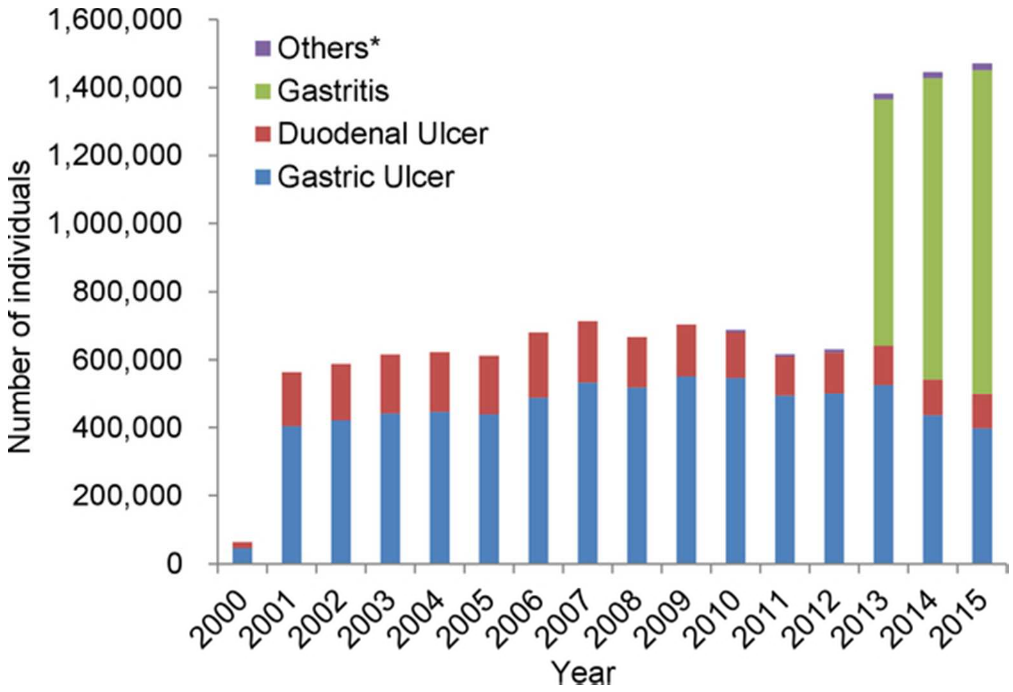

Figure 1. Annual number of individuals who had successful eradication (both first-line and second-line).
*Others: gastric mucosa associated lymphoid tissue lymphoma, idiopathic thrombocytopenic purpura, and post-endoscopic resection of early gastric cancer.

53x35mm (300 x 300 DPI)

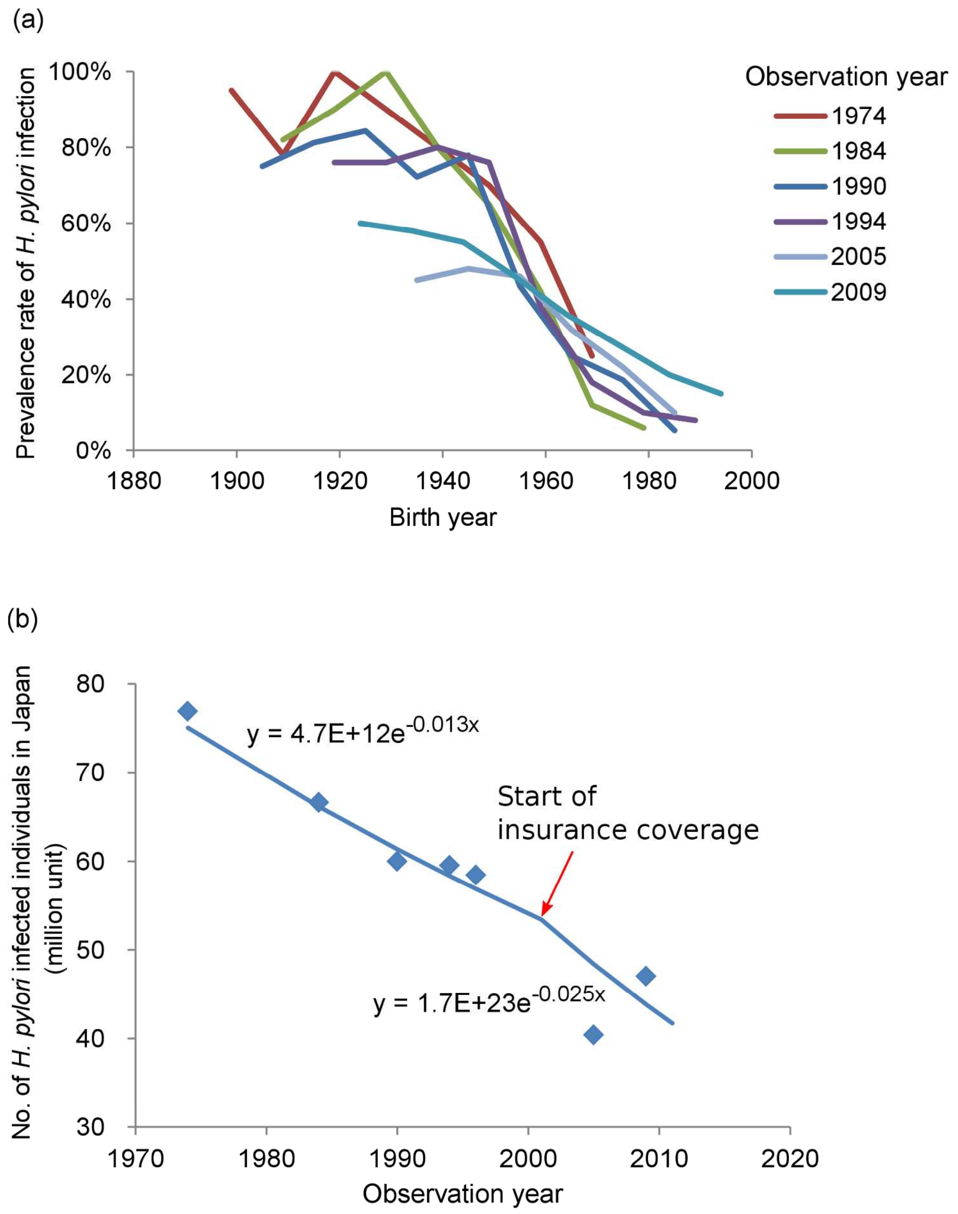

Figure 2. Trends in (a) the percentages of *H. pylori* infection by birth year based on previous studies and (b) the nationwide number of infected individuals as estimated based on the previous studies. *Data from the study by Watabe et al.[31] were excluded in (a) as that study divided the age group into 2 age groups: below and above age 60. *H. pylori, Helicobacter pylori.*

746x951mm (72 x 72 DPI)

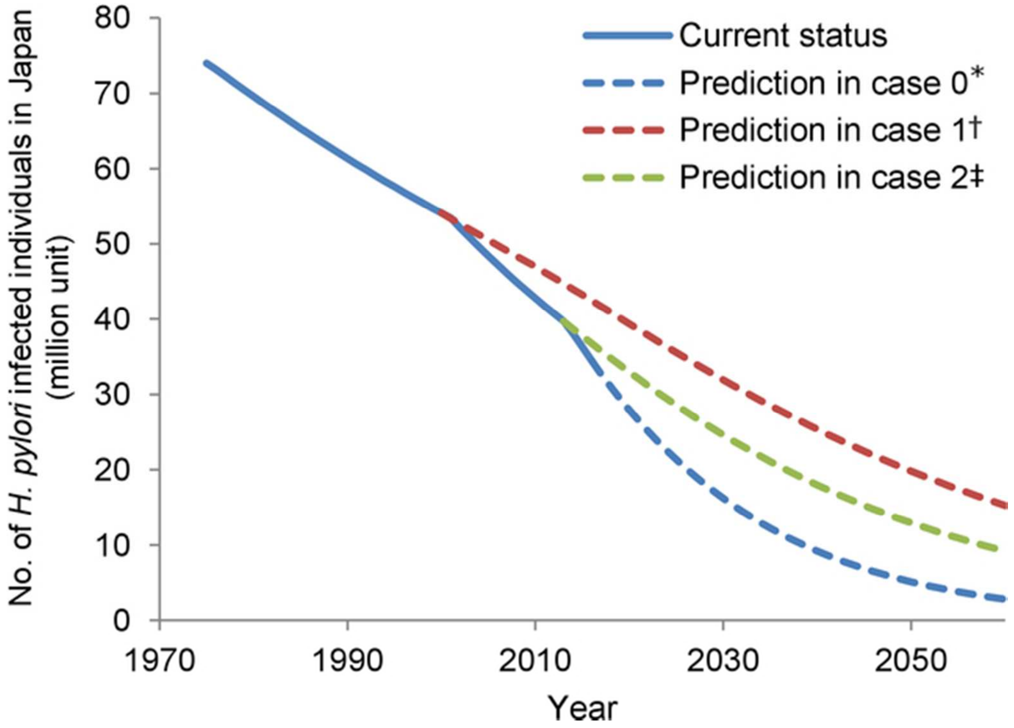

Figure 3. Trend and prediction of the number of *H. pylori* infected individuals. *Case 0, in current policy; †case 1, if policy change had not occurred in 2000; ‡case 2, if policy change had not occurred in 2013. *H. pylori, Helicobacter pylori*.

54x38mm (300 x 300 DPI)

For peer review only - http://bmjopen.bmj.com/site/about/guidelines.xhtml

Supplementary information

Table S1. The number of primary eradication individuals, mean age, and percentage of males for each year in the JMDC and MDV databases.

		2005	2006	2007	2008	2009	2010	2011	2012	2013	2014	2015
JMDC	Number	1,001	987	1,049	1,426	2,078	3,231	3,985	4,649	19,341	21,951	21,421
	Mean age	41.5	41.1	40.4	39.2	38.4	37.7	38.4	38.3	35.8	37.1	35.9
	Male %	83	79	77	70	67	66	65	65	58	80	58
MDV	Number	-	-	-	442	2,108	5,655	7,520	9,588	36,811	52,920	55,949
	Mean age	-	-	-	58.1	57.9	59.0	59.0	59.3	60.6	60.9	61.0
	Male %	-	-	-	65	66	64	64	64	57	55	55

JMDC, Japan Medical Data Center; MDV, Medical Data Vision.

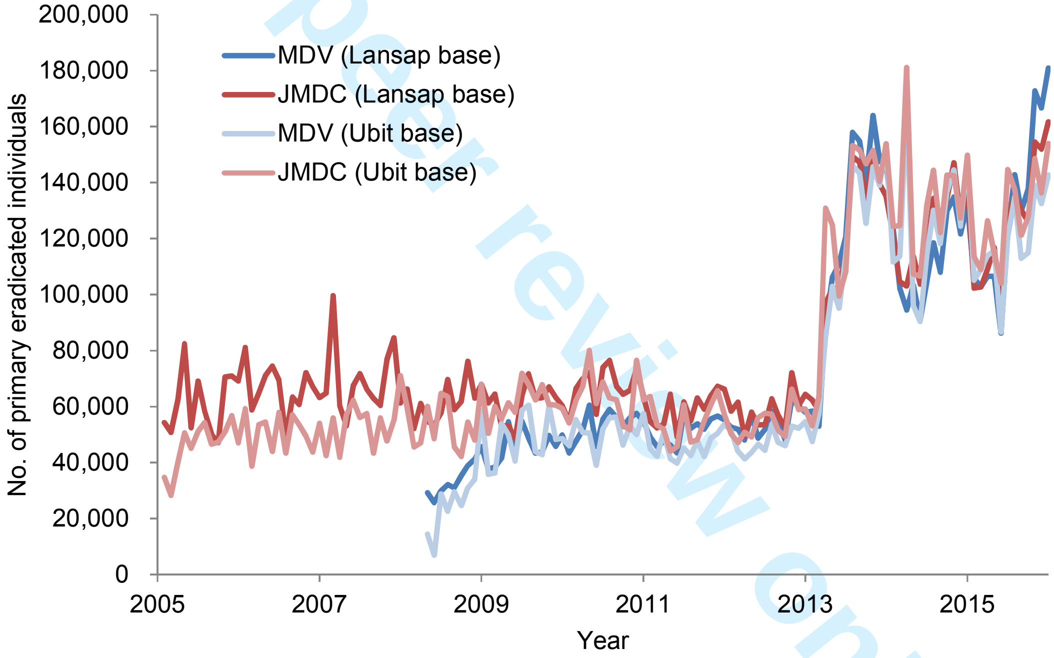

Figure S1. The number of individuals with the primary eradication of *H. pylori* according to each database.

The RECORD statement – checklist of items, extended from the STROBE statement, that should be reported in observational studies using routinely collected health data.

	Item No.	STROBE items	Location in manuscript where items are reported	RECORD items	Location in manuscript where items are reported
**Title and abstract**					
	1	(a) Indicate the study's design with a commonly used term in the title or the abstract (b) Provide in the abstract an informative and balanced summary of what was done and what was found	Title and Abstract; page 1–2.	RECORD 1.1: The type of data used should be specified in the title or abstract. When possible, the name of the databases used should be included.<br><br>RECORD 1.2: If applicable, the geographic region and timeframe within which the study took place should be reported in the title or abstract.<br><br>RECORD 1.3: If linkage between databases was conducted for the study, this should be clearly stated in the title or abstract.	Mentioned the type of data in Title and Abstract; page 1–2.<br><br>Reported the region in Title and Abstract; page 1–2.<br><br>N/A
**Introduction**					
Background rationale	2	Explain the scientific background and rationale for the investigation being reported	The 1st, 2nd and 3rd paragraphs of Introduction; page 5–7.		
Objectives	3	State specific objectives, including any prespecified hypotheses	The 4th paragraph (last paragraph) of Introduction; page 7.		
**Methods**					
Study Design	4	Present key elements of study design early in the paper	Methods (Study design section); page 9.		
Setting	5	Describe the setting, locations, and relevant dates, including periods of recruitment, exposure, follow-up, and data collection	Methods (Data sources section) ; page 7–8.		

For peer review only

Participants	6	*(a) Cohort study* - Give the eligibility criteria, and the sources and methods of selection of participants. Describe methods of follow-up<br>*Case-control study* - Give the eligibility criteria, and the sources and methods of case ascertainment and control selection. Give the rationale for the choice of cases and controls<br>*Cross-sectional study* - Give the eligibility criteria, and the sources and methods of selection of participants<br><br>*(b) Cohort study* - For matched studies, give matching criteria and number of exposed and unexposed<br>*Case-control study* - For matched studies, give matching criteria and the number of controls per case		RECORD 6.1: The methods of study population selection (such as codes or algorithms used to identify subjects) should be listed in detail. If this is not possible, an explanation should be provided.<br><br>RECORD 6.2: Any validation studies of the codes or algorithms used to select the population should be referenced. If validation was conducted for this study and not published elsewhere, detailed methods and results should be provided.<br><br>RECORD 6.3: If the study involved linkage of databases, consider use of a flow diagram or other graphical display to demonstrate the data linkage process, including the number of individuals with linked data at each stage.	The 1st paragraph of Patient identification and statistical analysis section in Methods; page 9–10.<br><br>Provided the detailed methods and results in the 1st paragraph of Patient identification and statistical analysis section in Methods and Patient characteristics section in Results page 9–11, 14.<br><br>N/A
Variables	7	Clearly define all outcomes, exposures, predictors, potential confounders, and effect modifiers. Give diagnostic criteria, if applicable.	Methods (Patient identification and statistical analysis section); page 10–13.	RECORD 7.1: A complete list of codes and algorithms used to classify exposures, outcomes, confounders, and effect modifiers should be provided. If these cannot be reported, an explanation should be provided.	Methods (Patient identification and statistical analysis section); page 10–13.
Data sources/ measurement	8	For each variable of interest, give sources of data and details of methods of assessment	Methods (Patient identification and statistical analysis		

For peer review only

		(measurement). Describe comparability of assessment methods if there is more than one group	section); page 9–11. We used two types of claims databases and two types of product sales data; therefore, four types of combination were obtained and then the combinations were compared (Figure S1)		
Bias	9	Describe any efforts to address potential sources of bias	Discussion (The 2nd paragraph of Strengths and limitations of this study section); page 18–19.		
Study size	10	Explain how the study size was arrived at	Methods (The 1st paragraph of Patient identification and statistical analysis section); page 9–10.		
Quantitative variables	11	Explain how quantitative variables were handled in the analyses. If applicable, describe which groupings were chosen, and why	Methods (Patient identification and statistical analysis section); page 11–13.		
Statistical methods	12	(a) Describe all statistical methods, including those used to control for confounding<br>(b) Describe any methods used to examine subgroups and interactions<br>(c) Explain how missing data were addressed<br>(d) *Cohort study* - If applicable, explain how loss to follow-up was addressed	Methods (Patient identification and statistical analysis section); page 10–13.		

For peer review only

		Case-control study - If applicable, explain how matching of cases and controls was addressed<br>*Cross-sectional study* - If applicable, describe analytical methods taking account of sampling strategy<br>(e) Describe any sensitivity analyses			
Data access and cleaning methods		..		RECORD 12.1: Authors should describe the extent to which the investigators had access to the database population used to create the study population.<br><br>RECORD 12.2: Authors should provide information on the data cleaning methods used in the study.	Methods (Data sources section); page 7.<br><br>N/A
Linkage		..		RECORD 12.3: State whether the study included person-level, institutional-level, or other data linkage across two or more databases. The methods of linkage and methods of linkage quality evaluation should be provided.	N/A
**Results**					
Participants	13	(a) Report the numbers of individuals at each stage of the study (*e.g.*, numbers potentially eligible, examined for eligibility, confirmed eligible, included in the study, completing follow-up, and analysed)<br>(b) Give reasons for non-participation at each stage.<br>(c) Consider use of a flow diagram	Results (Patient characteristics section); page 14.	RECORD 13.1: Describe in detail the selection of the persons included in the study (*i.e.,* study population selection) including filtering based on data quality, data availability and linkage. The selection of included persons can be described in the text and/or by means of the study flow diagram.	Results (Patient characteristics section); page 14.
Descriptive data	14	(a) Give characteristics of study	Results (Patient		

		participants (*e.g.*, demographic, clinical, social) and information on exposures and potential confounders<br>(b) Indicate the number of participants with missing data for each variable of interest<br>(c) *Cohort study* - summarise follow-up time (*e.g.*, average and total amount)	characteristics section and Table S1); page 14.		
Outcome data	15	*Cohort study* - Report numbers of outcome events or summary measures over time<br>*Case-control study* - Report numbers in each exposure category, or summary measures of exposure<br>*Cross-sectional study* - Report numbers of outcome events or summary measures	Results (Trend in the number of individuals with the primary eradication therapy and successful eradication section); page 14.		
Main results	16	(a) Give unadjusted estimates and, if applicable, confounder-adjusted estimates and their precision (e.g., 95% confidence interval). Make clear which confounders were adjusted for and why they were included<br>(b) Report category boundaries when continuous variables were categorized<br>(c) If relevant, consider translating estimates of relative risk into absolute risk for a meaningful time period	Results (Trend in the number of individuals with the primary eradication therapy and successful eradication section and Figure 1, and Trend and prediction of H. pylori infection and the effect of insurance policy changes section and Figure 3); page 14–15.		
Other analyses	17	Report other analyses done—e.g., analyses of subgroups and interactions, and sensitivity	N/A		

		analyses			
**Discussion**					
Key results	18	Summarise key results with reference to study objectives	The 1st paragraph of Discussion; page 15–16.		
Limitations	19	Discuss limitations of the study, taking into account sources of potential bias or imprecision. Discuss both direction and magnitude of any potential bias	Discussion (The 2nd paragraph of Strengths and limitations of this study section); page 18-19.	RECORD 19.1: Discuss the implications of using data that were not created or collected to answer the specific research question(s). Include discussion of misclassification bias, unmeasured confounding, missing data, and changing eligibility over time, as they pertain to the study being reported.	Discussion (The 2nd paragraph of Strengths and limitations of this study section); page 18-19.
Interpretation	20	Give a cautious overall interpretation of results considering objectives, limitations, multiplicity of analyses, results from similar studies, and other relevant evidence	The 2nd, 3rd and 4th paragraphs of Discussion; page 16-17.		
Generalisability	21	Discuss the generalisability (external validity) of the study results	Discussion (The 2nd and 3rd paragraphs of Strengths and limitations of this study section) ;page 18-19.		
**Other Information**					
Funding	22	Give the source of funding and the role of the funders for the present study and, if applicable, for the original study on which the present article is based	Footnote (Funding); page 20.		
Accessibility of protocol, raw data, and programming code				RECORD 22.1: Authors should provide information on how to access any supplemental information such as the study protocol, raw data, or programming code.	N/A

For peer review only

*Reference: Benchimol EI, Smeeth L, Guttmann A, Harron K, Moher D, Petersen I, Sørensen HT, von Elm E, Langan SM, the RECORD Working Committee. The REporting of studies Conducted using Observational Routinely-collected health Data (RECORD) Statement. *PLoS Medicine* 2015; in press.

*Checklist is protected under Creative Commons Attribution (CC BY) license.

For peer review only

BMJ Open

Impact of health insurance coverage for *Helicobacter pylori* gastritis on the trends in eradication therapy in Japan: retrospective observational study and simulation study based on real-world data.

For peer review only

Journal:	*BMJ Open*
Manuscript ID	bmjopen-2017-015855.R1
Article Type:	Research
Date Submitted by the Author:	20-Apr-2017
Complete List of Authors:	Hiroi, Shinzo ; Graduate School of Medicine and Public Health, Kyoto University, Department of Pharmacoepidemiology; Takeda Pharmaceutical Company, Limited, Japan Medical Affairs<br>Sugano, Kentaro; Jichi Medical University, Department of Medicine<br>Tanaka, Shiro; Graduate School of Medicine and Public Health, Kyoto University, Department of Pharmacoepidemiology<br>Kawakami, Koji; Graduate School of Medicine and Public Health, Kyoto University, Department of Pharmacoepidemiology
**Primary Subject Heading**:	Epidemiology
Secondary Subject Heading:	Gastroenterology and hepatology
Keywords:	health insurance, <i>Helicobacter pylori</i>, <i>Helicobacter pylori</i> eradication, <i>Helicobacter pylori</i>-positive gastritis

SCHOLARONE™
Manuscripts

Impact of health insurance coverage for *Helicobacter pylori* gastritis on the trends in eradication therapy in Japan: retrospective observational study and simulation study based on real-world data.

Shinzo Hiroi, HEOR Head[1,3], Kentaro Sugano, Professor Emeritus[2], Shiro Tanaka, Associate Professor[1], Koji Kawakami, Professor[1]

1 Department of Pharmacoepidemiology, Graduate School of Medicine and Public Health, Kyoto University, Kyoto, Japan

2 Department of Medicine, Jichi Medical University, Tochigi, Japan

3 Japan Medical Affairs, Takeda Pharmaceutical Company, Limited, Tokyo, Japan

Corresponding author:

Koji Kawakami

Department of Pharmacoepidemiology, Graduate School of Medicine and Public Health, Kyoto University, Yoshida Konoecho, Sakyoku, Kyoto 606-8501, Japan.

Tel: +81 75 753 9469

Fax: +81 75 753 4469

E-mail address: kawakami.koji.4e@kyoto-u.ac.jp

Keywords: health insurance, *Helicobacter pylori*, *Helicobacter pylori* eradication, *Helicobacter pylori*-positive gastritis

For peer review only

For peer review only - http://bmjopen.bmj.com/site/about/guidelines.xhtml

Abstract

Objectives

To explore the prevalence of *Helicobacter pylori (H. pylori)* infection in Japan and the trends of its eradication therapy before and after the changes of the insurance coverage policy, first started in 2000, and expanded to cover *H. pylori*-positive gastritis in 2013. The impacts the changes brought were estimated.

Methods

In this retrospective observational study and simulation study based on health insurance claims data, product sales data, and relevant studies, individuals who received triple therapy (amoxicillin, clarithromycin, proton-pump inhibitors or potassium-competitive acid blockers) were defined as the first-time patients for *H. pylori* eradication in two Japanese health insurance claims databases (from approximately 1.6 million and 10.5 million individuals). The each sales data of eradication packages and examination kits were used to estimate the number of *H. pylori*−eradicated individuals nationwide. The prevalence of *H. pylori* infection, including the future rate, was predicted using previous studies and the estimated population trend by a national institute. Cases completed prior to the policy change on insurance coverage were simulated to estimate what would have happened had there been no change in the policy.

Results

The numbers of patients first received eradication therapy were 81,119 and 170,993 from two databases. The nationwide estimated number of patients successfully eradicated was approximately 650,000 per year between 2001 and 2012, while it rapidly rose to 1,380,000 per year in 2013. The estimated prevalence of infection in 2050 is 5%; this rate was estimated to be 28% and 22% if the policy changes had not occurred in

For peer review only - http://bmjopen.bmj.com/site/about/guidelines.xhtml

2000 and 2013, respectively.

Conclusions

The impact of policy changes for *H. pylori* eradication therapy on the prevalence of infection was shown. The results suggest that insurance coverage expansion may also reduce the prevalence in other countries with a high prevalence of *H. pylori* infection if the reinfection is low.

For peer review only

For peer review only - http://bmjopen.bmj.com/site/about/guidelines.xhtml

Strengths and limitations of this study

- Demonstrates for the first time the impact of insurance policy expansion for *H. pylori* eradication therapy in a quantitative manner based on an analysis of nationwide real world data.
- Robust and reliable results were obtained from combinations of large-scale insurance claims databases and sales data of the most commonly used eradication treatments and test kits.
- The success rate of eradication was obtained from previous studies; therefore, the rate might be different from current clinical practice.
- The health insurance claims databases have potential biases: in one database, the information on individuals older than 65 years is limited because it is the information from employed individuals and their family members; another database included the data only from large hospitals.

For peer review only - http://bmjopen.bmj.com/site/about/guidelines.xhtml

Introduction

Throughout the world, gastric cancer is one of the most common cancers; 952,000 new patients were diagnosed in 2012.[1] The incidence of gastric cancer is higher in Asian countries; Korea, Japan, and China have the 1st, 3rd, and 5th highest rates, respectively, in the world.[2] In Japan, the prevalence and mortality of gastric cancer are constantly among the top three of all cancers. Therefore, it is considered to be one of the highest priorities in preventive policy. *Helicobacter pylori (H. pylori)* can cause gastric inflammation, which can then lead to gastric and duodenal ulcers, as well as gastric cancer.[3-5] Thus eradication of *H. pylori* is considered as an effective therapy in reducing the risk of those diseases.

Due to the concern of high gastric cancer prevalence in East Asian countries, some preventive programs have been launched to reduce the incidence of gastric cancer. In Korea, a cancer screening program was established by the government to provide for almost all people of eligible age (40 years or older for gastric cancer) with free screening or provision at minimum cost in 1999.[6] Large clinical trials and health economic studies have been conducted in China, and a consensus statement was formulated to encourage *H. pylori* eradication therapy.[6, 7] In Taiwan, the results of a community-level large screening and eradication program, as well as a health economic evaluation, support the efficacy of *H. pylori* eradication therapy.[8] In Japan, in November 2000, based on the results of diverse clinical studies,[3, 9-20] the government approved the addition of *H. pylori* eradication therapy in their insurance policy as a treatment for *H. pylori*-positive gastric ulcer and duodenal ulcer. Furthermore, insurance coverage was expanded in June 2010 to include gastric mucosa-associated lymphoid tissue lymphoma, idiopathic thrombocytopenic purpura, and post-endoscopic resection of early gastric

For peer review only - http://bmjopen.bmj.com/site/about/guidelines.xhtml

cancer, and in February 2013, to include *H. pylori*-positive gastritis based on the recommendations of Japanese guideline.[21]

Japanese health insurance is a system of universal coverage; the effect of change in health insurance coverage policy is spread throughout the nation. In terms of *H. pylori* eradication, everyone diagnosed as a disease covered for eradication therapy by health insurance can receive eradication therapy with coverage. Therefore, health insurance reimbursement seems to have the same or greater impact on clinical practice as recommendations from diagnostic/treatment guidelines in countries where universal health insurance coverage is established, such as in Japan and Korea. Various sizes of preventative programs for gastric cancer have been implemented in the high prevalence countries for both gastric cancer and *H. pylori* infection. In some countries, *H. pylori* eradication therapy for patients with *H. pylori*-positive gastric ulcer and duodenal ulcer has been covered by national health insurance. However, eradication therapy for *H. pylori*-positive gastritis has not been covered to date in these countries other than Japan.[8] The effect of insurance coverage expansion on the prevalence of *H. pylori* infection has been evaluated in only a few studies at the community level in Japan.[22,23] Nonetheless, the national-level prevalence rate of *H. pylori* infection has not been reported and its change has not been assessed after the insurance coverage for *H. pylori* eradication therapy was expanded to include *H. pylori*-positive gastritis in 2013.The progressive insurance expansion was reported to be efficient[24] and the incidence of peptic ulcer has decreased since the change in insurance coverage policy for *H. pylori* eradication in 2000.[25] They also estimated that gastric cancer mortality would decrease based on the assumption that 50% of *H. pylori*-infected patients would receive eradication therapy.[26] However, this estimate was based on neither the observed number

For peer review only - http://bmjopen.bmj.com/site/about/guidelines.xhtml

of patients undergoing *H. pylori* eradication nor the prevalence rate of infection. To evaluate the impact of changes to the insurance policy on the incidence of various diseases, including gastric cancer, it is necessary to elucidate the national trend of eradication therapy and the prevalence rate of infection before and after the changes in the insurance policy.

The primary objective of this study was to assess how health insurance policy changes have impacted eradication therapy and the prevalence rate of *H. pylori* infection in Japan. Furthermore, the future effect, as a result of the policy changes, on the prevalence of *H. pylori* infection was evaluated. In this study, health insurance claims databases and product sales data were used to estimate the number of eradication treatments. The successful eradication rate from 2000 onwards, at which time the health insurance began its coverage for the eradication therapy, was also estimated. The prevalence rate of infection and the number of infected individuals up to 2060 were predicted as based on the above data analysis and the prevalence rate of *H. pylori* infection as reported in previous studies. Furthermore, a simulation was conducted to estimate what the probable effects would be had the policy changes not been made.

Methods

Data sources

Insurance claims databases from Japan Medical Data Center (JMDC) from January 2005 to December 2015 and Medical Data Vision (MDV) from April 2008 to December 2015 were used for the analyses. The JMDC database is a registry of health insurance claims and medical examination records for insured individuals and their families in more than 50 health insurance societies. Because this database only included

For peer review only - http://bmjopen.bmj.com/site/about/guidelines.xhtml

For peer review only

information on company employees and their families, the information for those older than 65 years was limited. Also, there were no data for those older than 75 years. Until 2014, this database covered 1.6 million individuals which accounted for 1% of the Japanese population. The medical database from MDV covered 10.5 million individuals in 192 acute care hospitals using diagnostic procedure combination/per-diem payment system (DPC/PDPS). It included 11% of acute care hospitals in Japan with the number of beds from 20 to more than 1,000. These databases included the patient's gender, age, diagnosis, prescription information, and so on. Diagnosis information is based on the International Classification of Diseases 10th Revision, and drugs are coded in the Anatomical Therapeutic Chemical Classification System. Both databases included anonymous and personally unidentifiable data.

To estimate the nationwide number of infected individuals, product sales data for the most common eradication medicine, and test kit for *H. pylori* infection were analyzed. The sales data for eradication medicine, Lansap® (Takeda Pharmaceutical Co., Ltd.), which consists of lansoprazole, amoxicillin, and clarithromycin in one package was provided by the manufacturer from December 2002 through December 2015. The data for ^{13}Urea Breath Test (UBIT®, Otsuka Pharmaceutical Co., Ltd.) was obtained as well from November 2000 through December 2015.

To determine the trend in the number of *H. pylori* infected individuals, previously published Japanese studies were used (Table 1).

Table 1. Studies on the number of *H. pylori* infected individuals and prevalence rate of infection

First Author	Number of Subjects	Population	Study Design	Observation Year
Asaka M[27]	426	Asymptomatic children, students and adults (participating at the health screening center) living in Sapporo, Hokkaido	Observational study	1990

For peer review only - http://bmjopen.bmj.com/site/about/guidelines.xhtml

Fujisawa T[28]	349 (1974)<br>324 (1984)<br>342 (1994)	Healthy persons living in 7 prefectures in the central part of Japan	Observational study	1974<br>1984<br>1994
Watabe H[29]	6,983	Participants in a mass health appraisal programme.	Observational study	1996
Ueda J[30]	14,716	Individuals who underwent a health checkup in 7 prefectures (Hokkaido, Aomori, Yamagata, Gunma, Aichi, Shiga and Kagawa)	Observational study	2005
Shiota S[31]	5,550	Patients of Oita University Hospital, Oita, Japan	Observational study	2009

Study design

This study is a retrospective observational study and simulation study based on the health insurance claims data, product sales data, and relevant published studies. The steps were as follows; firstly, the number of individuals who received the eradication therapy and those who had successful eradication were estimated based on the analyses of the health insurance claims databases and product sales data. Secondly, the trend in the number of *H. pylori* infected individuals was determined from previously published studies. Thirdly, the prevalence rate and trend of *H. pylori* infection was estimated and forecasted from the results of the first and second step. Finally, to fully evaluate the impact of the policy changes, a simulation was made considering effects which likely would have occurred without the insurance policy changes in 2000 and 2013.

Patient identification and analysis

In the JMDC and MDV databases, the individuals who received triple therapy, either the primary eradication package (such as Lansap® and other packaged products), or the combination of amoxicillin, clarithromycin, and either proton-pump inhibitors or potassium-competitive acid blockers (all prescribed within the same month), were defined as individuals with primary eradication of *H. pylori*. The drugs used for the

For peer review only - http://bmjopen.bmj.com/site/about/guidelines.xhtml

therapy were defined by product name in JMDC database and remuneration code in MDV database. The examination was defined by remuneration code, and the diagnosis for those who had eradicated was defined by name of diagnosis in both databases. To estimate the number of individuals who received primary eradication therapy in the nation, the following were calculated in these databases:

1) Percentage of individuals who used Lansap® for primary eradication therapy to all individuals who received the primary eradication.
2) Percentage of individuals who received UBIT® for the *H. pylori* test after eradication to all individuals who took the *H. pylori* test.

To calculate the number of individuals who achieved successful eradication, the following were assumed (Figure 1):

- The primary and secondary success rates of eradication for this study were presumed to be 75% and 90%, respectively, based on previous studies in Japan.[25, 27] Secondary eradication was premised to be performed for all those who failed primary eradication. Therefore, the success rate of the eradications was estimated to be 98% of the primary eradication, and the percentage was used as the success rate in this study.
- New infection in adulthood was reported to be rare[32] and reinfection per year after the eradication therapy in Japan is reported to be approximately 1%;[33,34] however it was assumed to be 0% in this study.

The nationwide number of individuals who received primary eradication was estimated based on 1) and 2) with sales data of Lansap® and UBIT® as follows:

- The monthly number of individuals who received primary eradication from January 2010 was calculated as the mean of four estimates (Lansap®-base from

For peer review only - http://bmjopen.bmj.com/site/about/guidelines.xhtml

MDV and JMDV, UBIT®-base from MDV and JMDC).

- The monthly number of individuals who received primary eradication from January 2006 to December 2009 was calculated as the mean of two estimates from the JMDC database (Lansap®-base and UBIT®-base).
- The monthly number of individuals who received primary eradication from November 2000 to December 2005 was extrapolated using the sales number of UBIT® in each month and the UBIT® share rate in 2006 on the assumption that the share rate in this period was the same as that in 2006.

The formula used is as follows:

$(\text{Successful Eradication Number})_{YM}$ = $(\text{Primary Successful Eradication Number})_{YM}$ + $(\text{Secondary Successful Eradication Number})_{YM}$

$(\text{Primary Successful Eradication Number})_{YM}$ = $(\text{Primary Eradication Number})_{YM}$ × (Primary Eradication Success Rate, 75%)

$(\text{Secondary Successful Eradication Number})_{YM}$ = [$(\text{Primary Eradication Number})_{YM}$ - $(\text{Primary Successful Eradication Number})_{YM}$] × (Secondary Eradication Success Rate, 90%)

$(\text{Primary Eradication Number})_{YM}$ = [$(\text{Primary Eradication Number with Lansap®})_{YM,JMDC}$ + $(\text{Primary Eradication Number with Lansap®})_{YM,MDV}$ + $(\text{Primary Eradication Number with UBIT®})_{YM,JMDC}$ + $(\text{Primary Eradication Number with UBIT®})_{YM,MDV}$] / 4

$(\text{Primary Eradication Number with Lansap®})_{YM,Database}$ = $(\text{Sales Number of Lansap®})_{YM}$ / $(\text{Share of Lansap®})_{YM,Database}$

$(\text{Primary Eradication Number with UBIT®})_{YM,Database}$ = $(\text{Examination Number})_{YM,Database}$ × $(\text{Ratio of Primary Eradication to Total Examination})_{YM,Database}$

For peer review only - http://bmjopen.bmj.com/site/about/guidelines.xhtml

$(\text{Examination Number})_{YM,Database} = (\text{Sales Number of UBIT®})_{YM} / (\text{Share of UBIT®})_{YM,Database}$

where YM is year month, and Database = JMDC or MDV database.

The number of infected individuals and prevalence rate of infection were estimated based on the previous Japanese studies shown in Table 1. The number of infected individuals until March 2013 was estimated from previous studies[27-31] and vital statistics in Japan conducted by Ministry of Health, Labour and Welfare.[35] An exponential decay approximation curve was calculated based on the results of the previous studies until 2000. After 2000, the estimated mean monthly number of individuals who achieved successful eradication from January 2001 to March 2013 was taken into account. The number of infected individuals from April 2013 was estimated using the estimated mean monthly number of individuals who achieved successful eradication from April 2013 to December 2015, giving consideration to the decrease in the number of infected individuals due to death. It was calculated as follows:

$$(\text{Infection Number})_{CY} = \frac{\sum_{DOBYR} \text{National Population}_{DOBYR,\ CY} \times \text{Prevalence Rate}_{DOBYR,\ CY}}{\sum_{DOBYR} \text{National Population}_{DOBYR,\ CY}}$$

where $(\text{Prevalence Rate})_{DOBYR,CY}$ is the prevalence rate of *H. pylori* infection by birth year in each observation year calculated in this study, and $(\text{National Population})_{DOBYR,CY}$ is the population by birth year in each observation year; where DOBYR is birth year by 5 years, and CY is the calendar year of observation of each study. An exponential parameter that minimizes the sum of squared distances from $(\text{Infection Number})_{CY}$ was calculated (least squares method), assuming the decrease of the number of *H. pylori* infected individuals by any reason other than eradication (*i.e.,* aging) followed an exponential function and the decrease of that by eradication was constant from 2001

For peer review only - http://bmjopen.bmj.com/site/about/guidelines.xhtml

through 2013.

Assuming that *H. pylori* infection would be decreasing exponentially after 2015 (by consideration of the natural decrease due to the death of older infected individuals), a simulation was performed using the number of infected individuals obtained in the previous section and the population forecast from the National Institute of Population and Social Security Research[36] to predict the number of infected individuals in the future. The prevalence rate of infection was also simulated for the case of no policy change regarding insurance coverage in 2000 and 2013. This was simulated as follows:

1) The number of *H. pylori* infected individuals calculated in the previous section was taken to be $(\text{Infection Number})_{CY}$ before 2013.
2) The number of *H. pylori* infected individuals from 2013 through 2015 was assumed to be decreased from $(\text{Infection Number})_{CY}$ before 2013, based on the estimated number of individuals who achieved successful eradication by the analysis of the JMDC and MDV databases and sales data of drugs.
3) If Case 0, $(\text{Prevalence Rate})_{CY,case} = (\text{Infection Number})_{CY} / (\text{National Population})_{CY}$, in $1975 \leq CY \leq 2000$; or $(\text{Prevalence Rate})_{CY,case} / (\text{Prevalence Rate})_{CY-1,case} = (\text{Infection Number})_{CY-1} / (\text{Infection Number})_{CY-2}$, in $2016 \leq CY$.
4) If Case 1, $(\text{Prevalence Rate})_{CY,case} = (\text{Infection Number})_{CY} / (\text{National Population})_{CY}$, in $1975 \leq CY \leq 2000$;or $(\text{Prevalence Rate})_{CY,case} / (\text{Prevalence Rate})_{CY-1,case} = (\text{Infection Number})_{CY-1} / (\text{Infection Number})_{CY-2}$ in $2001 \leq CY$.
5) If Case 2, $(\text{Prevalence Rate})_{CY,case} = (\text{Infection Number})_{CY} / (\text{National Population})_{CY}$, in $1975 \leq CY \leq 2012$;or $(\text{Prevalence Rate})_{CY,case} / (\text{Prevalence Rate})_{CY-1,case} = (\text{Infection Number})_{CY-1} / (\text{Infection Number})_{CY-2}$ in $2013 \leq CY$.

For peer review only - http://bmjopen.bmj.com/site/about/guidelines.xhtml

Case 0 is with the current policy; Case 1 or 2 represent the case in which policy change had not occurred in 2000 or 2013.

Statistical analysis was carried out using Excel 2010 (Microsoft, Redmond, WA, USA) and SAS version 9.4 (SAS Institute, Cary, NC, USA).

Results

Patient characteristics

The total number of individuals who received primary eradication was 81,119 (mean age 36.8 years; males 61%) from the JMDC database, and 170,993 (mean age 60.6 years; males 57%) from the MDV database. The characteristics for each year are shown in Table S1.

Trend in the number of individuals with the primary eradication therapy and successful eradication

The difference among the four (Lansap®-base from MDV and JMDV, UBIT®-base from MDV and JMDC) estimated numbers of individuals who received primary eradication was confirmed to be minimal after 2010 (Figure S1). The nationwide number of individuals who had successful eradication (both first-line and second-line) was estimated as shown in Figure 2. The number was approximately 650,000 per year between 2001 and 2012, which has reached a steady state of approximately 700,000 per year after an increase in 2006. However, there was a slight decrease in 2011. It markedly increased to 1,380,000 in 2013, which is more than double the number observed in 2012. In the diagnoses for those who had successful eradication treatment (Figure 2), gastritis accounted for more than half of the diagnoses since 2013. The

For peer review only - http://bmjopen.bmj.com/site/about/guidelines.xhtml

average number of individuals who received successful eradication treatment up to March 2013 was 54,000 per month, while it was 124,000 per month after March 2013. The cumulative total of individuals who received successful eradication treatment was more than 10 million up to September 2014.

Trend in the number of infected individuals and the prevalence rate of infection

Figure 3a illustrates the prevalence rate of *H. pylori* infection by birth year from previous studies. This shows higher prevalence rates of infection in the cohorts with earlier birth years. Also, there was a tendency for the difference of prevalence rates of infection among studies to be larger in those with an earlier birth year, and the rate was lower in later observations. The overall estimated prevalence rate of infection was lower in later years (Figure 3b). The lines were fitted after taking the effect of insurance policy change in November 2011 into account, shown in Figure 3b.

Trend and prediction of H. pylori infection and the effect of insurance policy changes

The pattern of the number of infected individuals from 2016 in Japan was predicted (blue broken line in Figure 4) based on the trend in the number of infected individuals derived from the results above and the population forecast.[35] The patterns, in the case without policy changes in insurance coverage in 2000 and 2013, were also simulated (red and green broken lines in Figure 4). The simulation showed that the number of infected individuals would decrease to 16,200,000 individuals in 2030, or 14% of the population, and further decrease to 5% of the population in 2050. These figures would have been 28% and 21% in 2030 if the policy changes had not occurred in 2000 and 2013, respectively.

For peer review only - http://bmjopen.bmj.com/site/about/guidelines.xhtml

Discussion

This study described the status of eradication therapy and trend of *H. pylori* infection using large insurance claims databases that reflected actual clinical practice at the national level in Japan. The analysis showed that the prevalence rate of *H. pylori* infection has decreased after the approval to include eradication therapy in the insurance policy in 2000. The number of successful eradications more than doubled immediately after insurance coverage for *H. pylori* eradication therapy was expanded in 2013. The simulation indicated that the prevalence rate of *H. pylori* infection would decrease and reach approximately 14% in 2030 and 5.4% in 2050.

Although it is difficult to compare the prevalence rate of infection among studies due to time and sample difference, it is worthwhile to compare the rate with other countries. The prevalence rate of *H. pylori* infection varies markedly in different countries; in general, it is higher in developing countries and lower in developed countries.[37] The prevalence rate of infection was reported to be 92% in Bangladesh,[38] 75% in Vietnam,[39] 41-72% in China,[40] and 54-60% in Korea,[41,42] while it was 15-22% in Australia[43,44] and 8-27% in the USA.[45,46] Although the estimate of the prevalence rate of *H. pylori* infection in our study was 43% in Japan in 2000, based on our simulation, the prevalence rate of infection in Japan in 2030 with expanded insurance coverage would be almost the same as the Australian rate (15%), and it would reach the North American level (8-27%) by approximately 2050.

The estimate from the previous studies indicated a higher prevalence rate of infection in older cohorts, which can be explained by environmental factors such as poor sanitation.[47,48] It has also been suggested that nowadays *H. pylori* infection occurs

For peer review only - http://bmjopen.bmj.com/site/about/guidelines.xhtml

in childhood in Japan.[49,50] As a result, the number of infections would have naturally decreased even without eradication therapy due to the death of older infected individuals. However, our simulation showed that the prevalence rate of infection would have been higher than 14% and 7% in 2030 if the policy changes had not occurred in 2000 and 2013 respectively. It was therefore evident that those insurance policy changes had contributed to the reduction in the prevalence of *H. pylori* infection. Japan has established a universal health insurance coverage system, which means that by law all Japanese residents are entitled to health insurance coverage for medical treatments. This system has a great impact on the dissemination of medical treatments; consequently the use of eradication therapy is highly influenced by the presence of the health insurance and coverage for diseases.

The results of this study could be a good indicator for the implementation of insurance coverage for eradication of *H. pylori* in countries where *H. pylori* is prevalent, especially the East Asian countries. Countries and regions, such as Korea, China, and Taiwan, have been conducting clinical trials of *H. pylori* eradication; however, they have not yet established any policy for *H. pylori* eradication. This study is likely to be used as one of the references in considering effect on policy change in such countries. This study might also be important in providing direction for future research in Japan. In 2016, the revised edition of the Japanese Guideline for Diagnosis and Treatment for *H. pylori* infection was published following that of 2009. In the latest guideline, the expansion of insurance coverage for the treatment for *H. pylori* gastritis in 2013 is described.[51] Also, a regimen with potassium-competitive acid blocker (P-CAB)-based triple therapy, which is newly described in the guideline, demonstrated a high eradication rate compared to the conventional proton pump inhibitor-based triple

For peer review only - http://bmjopen.bmj.com/site/about/guidelines.xhtml

therapy.[51,52] It seems that the prevalence rate of infection could be further reduced in Japan.

Comparison with other studies

There have been some community level studies investigating the status of eradication in clinical practices in Japan.[22, 23] Nevertheless, to our knowledge, there was no study describing the status of eradication therapy at the national level. Asaka *et al.* have reported that the insurance policy change could increase the number of eradications, reduce the number of infected individuals, and decrease mortality from gastric cancer.[24,25] They estimated that the number of deaths from gastric cancer would reach 60,000 in 2020 without any countermeasures, while it would be half if 50% of infected individuals receive eradication therapy. However, this assumption was not based on actual observations. Our study, using real world data reflecting actual medical practice, showed a rapid increase in patients receiving *H. pylori* eradication, after the insurance policy change in 2013.

Strengths and limitations of this study

Here, a national level evaluation using real world data, allowed us to analyze the impact of insurance policy expansion for *H. pylori* eradication therapy in a quantitative manner. Furthermore, robust and reliable results were obtained from combinations of large-scale insurance claims databases and sales data of the most commonly used eradication treatments and test kits.

However, this study has several limitations. First, the success rate of eradication was obtained from previous studies;[25,27] thus, the rate might be different

For peer review only - http://bmjopen.bmj.com/site/about/guidelines.xhtml

from clinical practice, including the possibility that it may be estimated higher than the actual rate considering the effect of the increase in bacteria resistant to antibiotics. Nevertheless, the success rate in this study is believed to be close to the actual rate. Second, the health insurance claims databases have potential biases. The information available was limited for those older than 65 years in the JMDC database because it consisted of information on employed individuals and their family members. In addition, those who were self-employed and employees in small-to-medium-sized enterprises were not included. The information in the MDV database was obtained from the hospital using DPC/PDPS. Indeed, the mean ages of individuals who received primary eradication in both databases were different. However, the product share rates calculated in both databases were very similar after 2010, and the estimates of the number of individuals with the primary eradication were almost identical in both databases. Therefore, the impact of the different age distribution was believed to be minimal, and the estimates are believed to be accurate.

Despite these limitations, this study used the information from reliable clinical studies and large databases covering a significant number of Japanese citizens. Consequently, it is believed that the information presented here reflects the clinical status in Japan.

Conclusion and policy implications

This study described and forecasted the trend of *H. pylori* eradication therapy and assessed the impact of insurance policy change on the prevalence rate of *H. pylori* infection. It has demonstrated that the policy change was associated with a reduction in the prevalence rate of *H. pylori* infection in Japan. Furthermore it is expected to lead to

For peer review only - http://bmjopen.bmj.com/site/about/guidelines.xhtml

a reduction in the incidence of gastric cancer. We believe that nation-wide health insurance coverage for *H. pylori* eradication could reduce the prevalence rate of *H. pylori* infection and possibly lead to a reduction in the incidence of gastric cancer in other countries as well.

For peer review only

For peer review only - http://bmjopen.bmj.com/site/about/guidelines.xhtml

Acknowledgements

The authors thank Takeda's collaborator, Otsuka Pharmaceutical Co., Ltd. for providing sales data of urea breath test diagnostic kits (UBIT®). The authors thank Kosuke Iwasaki and Tomomi Takeshima, employees of Milliman Inc., for analyzing the database, and writing and editorial suppport, respectively, funded by Takeda Pharmaceutical Co., Ltd.

Contributors

SH, KS and KK contributed to the concept and design of the study.

SH contributed to acquisition of data.

SH contributed to the analysis.

SH, KS, and ST contributed to interpretation of data.

SH, KS, ST and KK contributed to the writing of the manuscript and critical revision of the manuscript.

All authors approved the final version of the manuscript.

KK is the guarantor of the article.

Funding

This study was sponsored by Takeda Pharmaceutical Co., Ltd.

Competing interests

SH is an employee of Takeda Pharmaceutical Co., Ltd.

KS has received research grants from Eisai, Daiichi Sankyo Pharm, and Takeda Pharmaceutical Co., Ltd. He has also received lecture fees from Astellas Pharma,

For peer review only - http://bmjopen.bmj.com/site/about/guidelines.xhtml

Fujifilm, and Takeda Pharmaceutical Co., Ltd.

ST has no personal interests to declare.

KK received research funds from Dainippon Sumitomo Pharma, Olympus, Stella Pharma, Medical Platform Co., Novartis Pharmaceutical K.K., Bayer, and Maruho; honorarium from Astellas, Daiichi Sankyo Pharm, Taisho Pharmaceutical, Eisai, Novartis Pharmaceutical K.K., Mitsubishi Tanabe Pharma, Takeda Pharmaceutical Co., Ltd, Sanofi K.K.; consult fees from Olympus, Kyowa Hakko Kirin, Kaken Pharmaceutical, and Otsuka Pharmaceutical Co., Ltd.

There are no patents, products in development or marketed products to declare, relevant to those companies.

Ethical approval

This study was approved by Ethic Committee, Kyoto University Graduate School and Faculty of Medicine (R0126-1). This study was exempted from obtaining individual informed consent based on Ethical Guidelines for Medical and Health Research Involving Human Subjects by Ministry of Education, Culture, Sports, Science and Technology, and Ministry of Health, Labour, and Welfare.

Provenance and peer review

Not commissioned; externally peer reviewed.

Data sharing statement

No additional data are available.

For peer review only

Transparency

The lead author (KK) affirms that the manuscript is an honest, accurate, and transparent account of the study reported; that no important aspects of the study have been omitted; and that any discrepancies from the study as planned have been explained.

This is an Open Access article distributed in accordance with the Creative Commons Attribution Non Commercial (CC BY-NC 3.0) license, which permits others to distribute, remix, adapt, build upon this work non-commercially, and license their derivative works on different terms, provided the original work is properly cited and the use is non-commercial. See: http://creativecommons.org/licenses/by-nc/3.0/.

For peer review only

References

1. Ferlay J, Soerjomataram I, Dikshit R, et al. Cancer incidence and mortality worldwide: sources, methods and major patterns in GLOBOCAN 2012. *Int J Cancer* 2015;136:E359-86 doi: 10.1002/ijc.29210.

2. World Cancer Research Fund International. Stomach cancer statistics. 2016. http://www.wcrf.org/int/cancer-facts-figures/data-specific-cancers/stomach-cancer-statistics.

3. Fukase K, Kato M, Kikuchi S, et al. Effect of eradication of *Helicobacter pylori* on incidence of metachronous gastric carcinoma after endoscopic resection of early gastric cancer: an open-label, randomised controlled trial. *Lancet* 2008;372:392-97 doi: 10.1016/S0140-6736(08)61159-9.

4. Marshall BJ, Armstrong JA, McGechie DB, et al. Attempt to fulfil Koch's postulates for pyloric *Campylobacter*. *Med J Aust* 1985;142:436-39.

5. Morris A, Nicholson G. Ingestion of *Campylobacter pyloridis* causes gastritis and raised fasting gastric pH. *Am J Gastroenterol* 1987;82:192-99.

6. IARC Working Group. *Helicobacter pylori* eradication as a strategy for preventing gastric cancer. 2014. https://www.iarc.fr/en/publications/pdfs-online/wrk/wrk8/Helicobacter_pylori_Eradication.pdf.

7. Liu WZ, Xie Y, Cheng H, et al. Fourth Chinese National Consensus Report on the management of *Helicobacter pylori* infection. *J Dig Dis* 2013;14:211-21 doi: 10.1111/1751-2980.12034.

8. Lee S-Y. Current progress toward eradicating *Helicobacter pylori* in East Asian countries: differences in the 2013 revised guidelines between China, Japan, and

For peer review only - http://bmjopen.bmj.com/site/about/guidelines.xhtml

South Korea. *World J Gastroenterol* 2014;20:1493-502 doi: 10.3748/wjg.v20.i6.1493.

9. Asaka M, Kato M, Sugiyama T, et al. Follow-up survey of a large-scale multicenter, double-blind study of triple therapy with lansoprazole, amoxicillin, and clarithromycin for eradication of *Helicobacter pylori* in Japanese peptic ulcer patients. *J Gastroenterol* 2003;38:339-47 doi: 10.1007/s005350300061.

10. Emilia G, Longo G, Luppi M, et al. *Helicobacter pylori* eradication can induce platelet recovery in idiopathic thrombocytopenic purpura. *Blood* 2001;97:812-14 doi: 10.1182/blood.V97.3.812.

11. Gasbarrini A, Franceschi F, Tartaglione R, et al. Regression of autoimmune thrombocytopenia after eradication of *Helicobacter pylori*. *Lancet* 1998;352:878-78 doi: 10.1016/S0140-6736(05)60004-9.

12. Hentschel E, Brandstatter G, Dragosics B, et al. Effect of ranitidine and amoxicillin plus metronidazole on the eradication of *Helicobacter pylori* and the recurrence of duodenal ulcer. *N Engl J Med* 1993;328:308-12 doi: 10.1056/NEJM199302043280503.

13. Leodolter A, Kulig M, Brasch H, et al. A meta‐analysis comparing eradication, healing and relapse rates in patients with *Helicobacter pylori*‐associated gastric or duodenal ulcer. *Aliment Pharmacol Ther* 2001;15:1949-58 doi: 10.1046/j.1365-2036.2001.01109.x.

14. Malfertheiner P, Megraud F, O'Morain C, et al. Current concepts in the management of *Helicobacter pylori* infection: the Maastricht III Consensus Report. *Gut* 2007;56:772-81 doi: 10.1136/gut.2006.101634.

15. Miwa H, Sakaki N, Sugano K, et al. Recurrent peptic ulcers in patients following

For peer review only - http://bmjopen.bmj.com/site/about/guidelines.xhtml

For peer review only

successful *Helicobacter pylori* eradication: a multicenter study of 4940 patients. *Helicobacter* 2004;9:9-16 doi: 10.1111/j.1083-4389.2004.00194.x.

16. Ogura K, Hirata Y, Yanai A, et al. The effect of *Helicobacter pylori* eradication on reducing the incidence of gastric cancer. *J Clin Gastroenterol* 2008;42:279-83 doi: 10.1097/01.mcg.0000248006.80699.7f.

17. Take S, Mizuno M, Ishiki K, et al. The effect of eradicating *Helicobacter pylori* on the development of gastric cancer in patients with peptic ulcer disease. *Am J Gastroenterol* 2005;100:1037-42 doi: 10.1111/j.1572-0241.2005.41384.x.

18. Takenaka R, Okada H, Kato J, et al. *Helicobacter pylori* eradication reduced the incidence of gastric cancer, especially of the intestinal type. *Aliment Pharmacol Ther* 2007;25:805-12 doi: 10.1111/j.1365-2036.2007.03268.x.

19. Wong BC-Y, Lam SK, Wong WM, et al. *Helicobacter pylori* eradication to prevent gastric cancer in a high-risk region of China: a randomized controlled trial. *JAMA* 2004;291:187-94 doi: 10.1001/jama.291.2.187.

20. Wotherspoon AC, Diss TC, Pan L, et al. Regression of primary low-grade B-cell gastric lymphoma of mucosa-associated lymphoid tissue type after eradication of *Helicobacter pylori*. *Lancet* 1993;342:575-77 doi: 10.1016/0140-6736(93)91409-F.

21. Asaka M, Kato M, Takahashi S, et al. Guidelines for the management of *Helicobacter pylori* infection in Japan: 2009 revised edition. *Helicobacter.* 2010;15:1-20 doi: 10.1111/j.1523-5378.2009.00738.x.

22. Hanamure Y, Honda A, Hokonohara A. [Eradication therapy in the era of *Helicobacter pylori* infection -Its issues-: Current status and issues of *Helicobacter pylori* treatment in Ichikikushikino-city (Kagoshima prefecture)]. *Helicobacter Research* 2014;18:74-77. Japanese.

For peer review only

For peer review only - http://bmjopen.bmj.com/site/about/guidelines.xhtml

23. Fujimori K. [The changes in *Helicobacter pylori* infection treatment: Is the number of infection reduced?]. *Helicobacter Research* 2014;18:314-319. Japanese.

24. Asaka M, Kato M, Sakamoto N. Roadmap to eliminate gastric cancer with *Helicobacter pylori* eradication and consecutive surveillance in Japan. *J Gastroenterol* 2014;49:1-8 doi: 10.1007/s00535-013-0897-8.

25. Sugano K, Osawa H, Satoh K. Clinical Management of *Helicobacter pylori*-The Japanese Perspective. *Dig Dis* 2014;32:281-89 doi: 10.1159/000357859.

26. Asaka M, Mabe K, Matsushima R, et al. *Helicobacter pylori* eradication to eliminate gastric cancer: the Japanese strategy. *Gastroenterol Clin North Am* 2015;44:639-48 doi: 10.1016/j.gtc.2015.05.010.

27. Asaka M, Kimura T, Kudo M, et al. Relationship of *Helicobacter pylori* to serum pepsinogens in an asymptomatic Japanese population. *Gastroenterology* 1992;102:760-66 doi: 10.1016/0016-5085(92)90156-S.

28. Fujisawa T, Kumagai T, Akamatsu T, et al. Changes in seroepidemiological pattern of *Helicobacter pylori* and hepatitis A virus over the last 20 years in Japan. Am J Gastroenterol 1999;94:2094-99 doi: 10.1111/j.1572-0241.1999.01283.x.

29. Watabe H, Mitsushima T, Yamaji Y, et al. Predicting the development of gastric cancer from combining *Helicobacter pylori* antibodies and serum pepsinogen status: a prospective endoscopic cohort study. Gut 2005;54:764-68 doi: 10.1136/gut.2004.055400.

30. Ueda J, Gosho M, Inui Y, et al. Prevalence of *Helicobacter pylori* infection by birth year and geographic area in Japan. Helicobacter 2014;19:105-10 doi: 10.1111/hel.12110.

31. Shiota S, Murakawi K, Suzuki R, et al. *Helicobacter pylori* infection in Japan.

For peer review only - http://bmjopen.bmj.com/site/about/guidelines.xhtml

Expert Rev Gastroenterol Hepatol 2013;7:35-40 doi: 10.1586/egh.12.67.

32. Cave DR. How is *Helicobacter pylori* transmitted? *Gastroenterology* 1997;113:S9-14 doi: 10.1016/S0016-5085(97)80004-2.

33. Take S, Mizuno M, Ishiki K, et al. Reinfection rate of *Helicobacter pylori* after eradication treatment: a long-term prospective study in Japan. *J Gastroenterol* 2012;47:641-6 doi: 10.1007/s00535-012-0536-9.

34. Okimoto T, Murakami K, Sato R, et al. Is the recurrence of *Helicobacter pylori* infection after eradication therapy resultant from recrudescence or reinfection, in Japan. *Helicobacter* 2003;8:186-91 doi: 10.1046/j.1523-5378.2003.00143.x.

35. Ministry of Health, Labour and Welfare. [Summary Report of Vital Statistics of Japan 2013 (final data)]. 2014. http://www.mhlw.go.jp/toukei/saikin/hw/jinkou/kakutei13/index.html. Japanese.

36. National Institute of Population and Social Security Research. [Future Population Prediction in Japan]. 2012. http://www.ipss.go.jp/syoushika/tohkei/newest04/sh2401top.html. Japanese.

37. Fock KM, Ang TL. Epidemiology of *Helicobacter pylori* infection and gastric cancer in Asia. *J Gastroenterol Hepatol* 2010;25:479-86 doi: 10.1111/j.1440-1746.2009.06188.x.

38. Ahmad MM, Rahman M, Rumi AK, et al. Prevalence of *Helicobacter pylori* in asymptomatic population--a pilot serological study in Bangladesh. *J Epidemiol* 1997;7:251-54 doi: 10.2188/jea.7.251.

39. Hoang TTH, Bengtsson C, Phung DC, et al. Seroprevalence of *Helicobacter pylori* infection in urban and rural Vietnam. *Clin Diagn Lab Immunol* 2005;12:81-85 doi: 10.1128/CDLI.12.1.81-85.2005.

For peer review only - http://bmjopen.bmj.com/site/about/guidelines.xhtml

For peer review only

40. Xie C, Lu NH. Review: clinical management of *Helicobacter pylori* infection in China. *Helicobacter* 2015;20:1-10 doi: 10.1111/hel.12178.

41. Yim JY, Kim N, Choi SH, et al. Seroprevalence of *Helicobacter pylori* in South Korea. *Helicobacter* 2007;12:333-40 doi: 10.1111/j.1523-5378.2007.00504.x.

42. Lim SH, Kwon JW, Kim N, et al. Prevalence and risk factors of *Helicobacter pylori* infection in Korea: nationwide multicenter study over 13 years. *BMC Gastroenterol* 2013;13:104. doi: 10.1186/1471-230X-13-104.

43. Moujaber T, MacIntyre CR, Backhouse J, et al. The seroepidemiology of *Helicobacter pylori* infection in Australia. *Int J Infect Dis* 2008;12:500-04 doi: 10.1016/j.ijid.2008.01.011.

44. Mitchell H1, Katelaris P. Epidemiology, clinical impacts and current clinical management of *Helicobacter pylori* infection. *Med J Aust* 2016;204:376-80.

45. Sonnenberg A, Lash RH, Genta RM. A national study of *Helicobactor pylori* infection in gastric biopsy specimens. *Gastroenterology* 2010;139:1894-901 doi: 10.1053/j.gastro.2010.08.018.

46. Cardenas VM, Mulla ZD, Ortiz M, et al. Iron deficiency and *Helicobacter pylori* infection in the United States. *Am J Epidemiol* 2006;163:127-34 doi: 10.1093/aje/kwj018

47. Goh KL, Chan WK, Shiota S, et al. Epidemiology of *Helicobacter pylori* infection and public health implications. *Helicobacter* 2011;16:1-9 doi: 10.1111/j.1523-5378.2011.00874.x.

48. Ueda M, Kikuchi S, Kasugai T, et al. *Helicobacter pylori* risk associated with childhood home environment. *Cancer Sc* 2003;94:914-8. doi: 10.1111/j.1349-7006.2003.tb01375.x

For peer review only

For peer review only - http://bmjopen.bmj.com/site/about/guidelines.xhtml

49. Konno M, Fujii N, Yokota S, et al. Five-year follow-up study of mother-to-child transmission of *Helicobacter pylori* infection detected by a random amplified polymorphic DNA fingerprinting method. *J Clin Microbiol* 2005;43:2246-50. doi: 10.1128/JCM.43.5.2246-2250.2005

50. Okuda M1, Miyashiro E, Booka M, et al. *Helicobacter pylori* colonization in the first 3 years of life in Japanese children. *Helicobacter* 2007;12:324-7. doi: 10.1111/j.1523-5378.2007.00510.x

51. The committee of the Japanese Society for Helicobacter Research. [Guidelines for diagnosis and treatment of *H. pylori* infection in Japan: 2016 revised edition]. Tokyo: Sentan Igaku-Sha; 2016. Japanese.

52. Murakami K, Sakurai Y, Shiino M, et al. Vonoprazan, a novel potassium-competitive acid blocker, as a component of first-line and second-line triple therapy for *Helicobacter pylori* eradication: a phase III, randomised, double-blind study. *Gut* 2016;65:1439-46 doi: 10.1136/gutjnl-2015-311304.

For peer review only

For peer review only - http://bmjopen.bmj.com/site/about/guidelines.xhtml

Figure legends

Figure 1. The model used to calculate the number of individuals with successful *H. pylori* eradication.

Figure 2. Annual number of individuals who had successful *H. pylori* eradication (both first-line and second-line). *Others: gastric mucosa associated lymphoid tissue lymphoma, idiopathic thrombocytopenic purpura, and post-endoscopic resection of early gastric cancer.

Figure 3. Trends in (a) the percentages of *H. pylori* infection by birth year based on previous studies and (b) the nationwide number of infected individuals as estimated based on the previous studies. *Data from the study by Watabe et al.[31] were excluded in (a) as that study divided the age group into 2 age groups: below and above age 60. *H. pylori, Helicobacter pylori.*

Figure 4. Trend and prediction of the number of *H. pylori* infected individuals. *Case 0, in current policy; †case 1, if policy change had not occurred in 2000; ‡case 2, if policy change had not occurred in 2013. *H. pylori, Helicobacter pylori.*

For peer review only

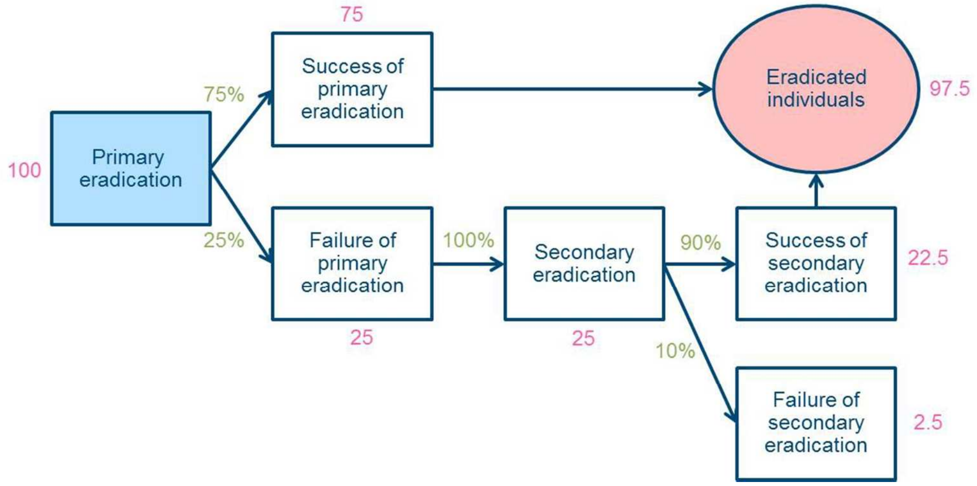

Figure 1. The model used to calculate the number of individuals with successful *H. pylori* eradication.

203x98mm (150 x 150 DPI)

For peer review only

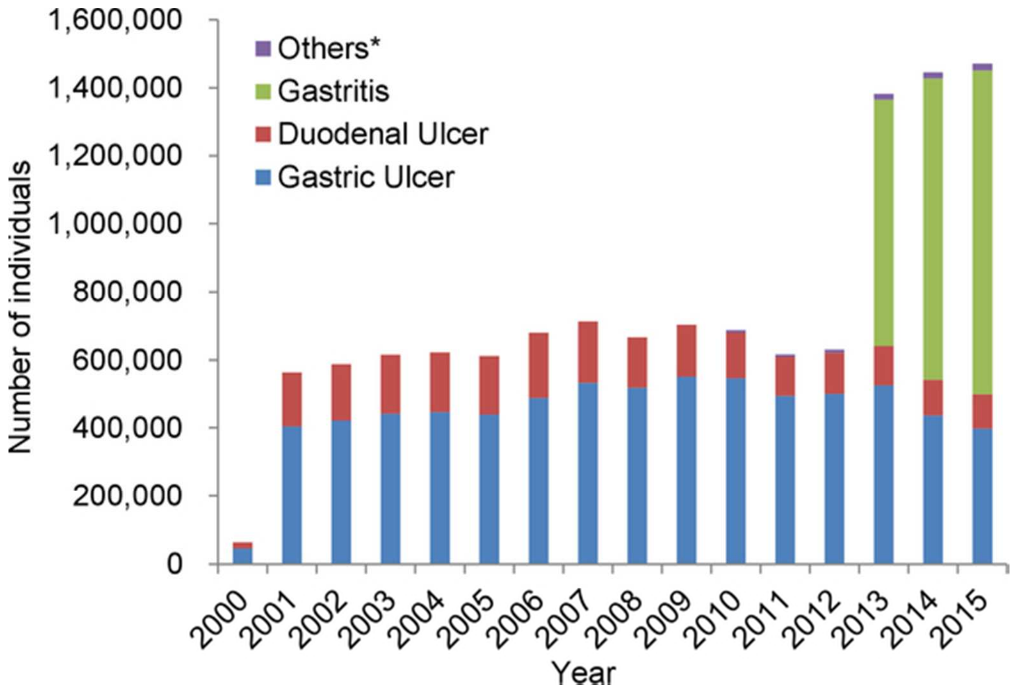

Figure 2. Annual number of individuals who had successful *H. pylori* eradication (both first-line and second-line). *Others: gastric mucosa associated lymphoid tissue lymphoma, idiopathic thrombocytopenic purpura, and post-endoscopic resection of early gastric cancer.

53x35mm (300 x 300 DPI)

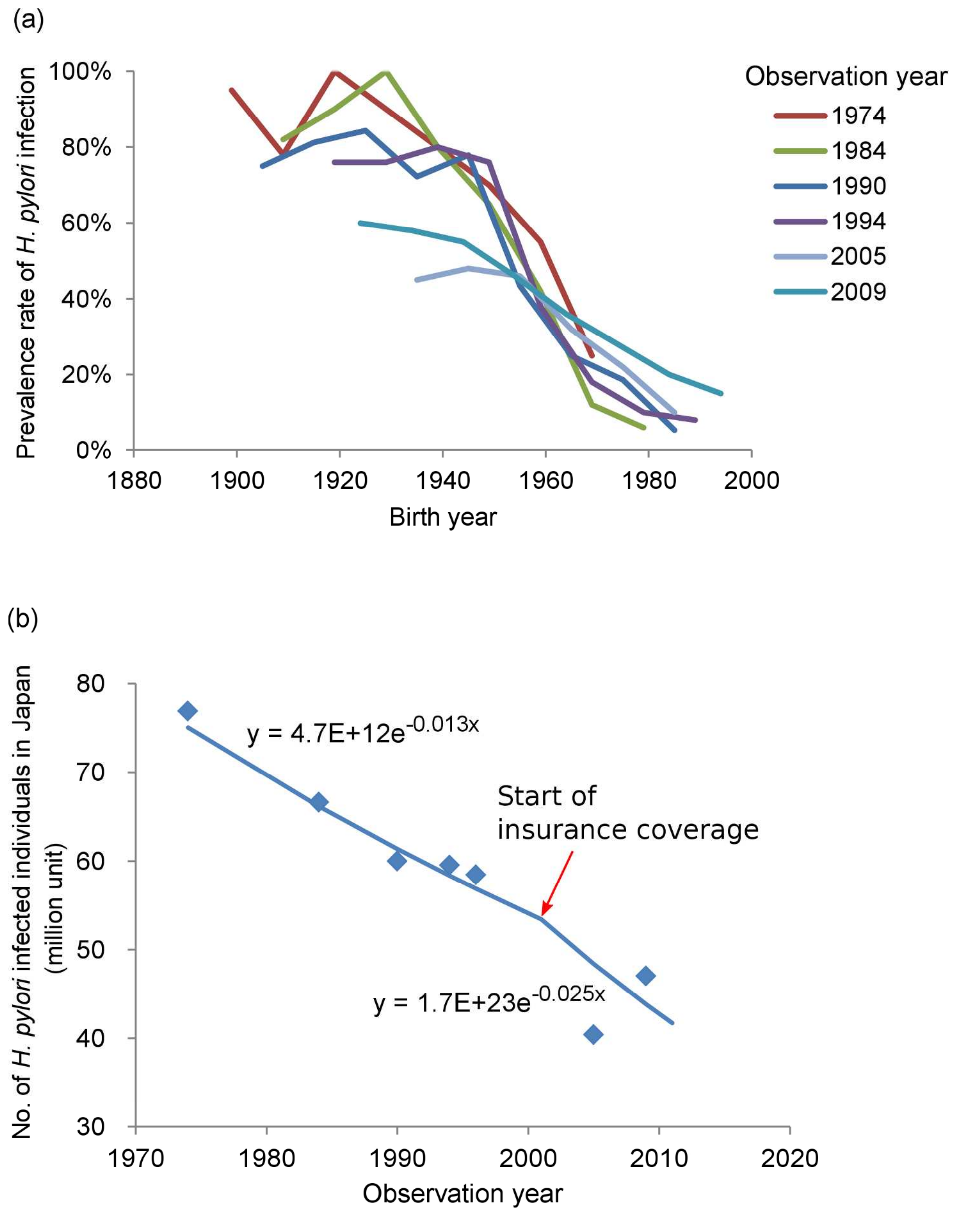

Figure 3. Trends in (a) the percentages of *H. pylori* infection by birth year based on previous studies and (b) the nationwide number of infected individuals as estimated based on the previous studies. *Data from the study by Watabe et al.31 were excluded in (a) as that study divided the age group into 2 age groups: below and above age 60. *H. pylori, Helicobacter pylori*.

746x951mm (72 x 72 DPI)

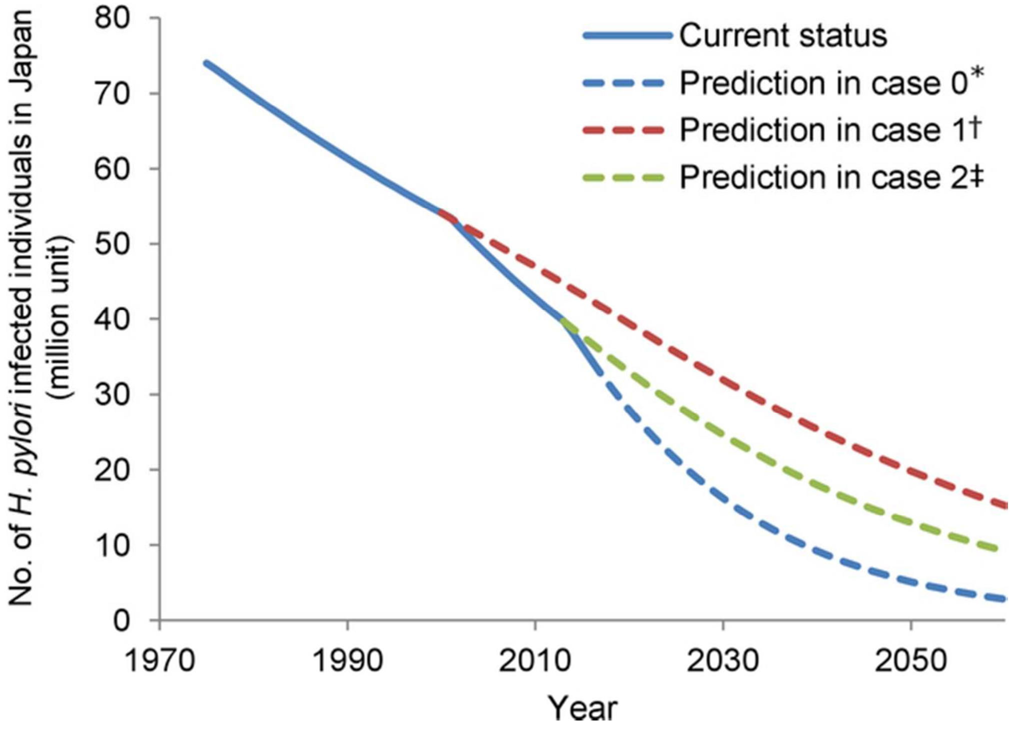

Figure 4. Trend and prediction of the number of *H. pylori* infected individuals. *Case 0, in current policy; †case 1, if policy change had not occurred in 2000; ‡case 2, if policy change had not occurred in 2013. *H. pylori, Helicobacter pylori*.

54x38mm (300 x 300 DPI)

ew only

Supplementary information

Impact of health insurance coverage for Helicobacter pylori gastritis on the trends in eradication therapy in Japan: retrospective observational study and simulation study based on real-world data.

Table S1. The number of primary eradication individuals, mean age, and percentage of males for each year in the JMDC and MDV databases.

		2005	2006	2007	2008	2009	2010	2011	2012	2013	2014	2015
JMDC	Number	1,001	987	1,049	1,426	2,078	3,231	3,985	4,649	19,341	21,951	21,421
	Mean age	41.5	41.1	40.4	39.2	38.4	37.7	38.4	38.3	35.8	37.1	35.9
	(SD)	(9.7)	(9.8)	(9.8)	(10.6)	(10.8)	(10.7)	(10.8)	(10.6)	(10.2)	(10.4)	(10.4)
	Male %	83	79	77	70	67	66	65	65	58	80	58
MDV	Number	-	-	-	442	2,108	5,655	7,520	9,588	36,811	52,920	55,949
	Mean age	-	-	-	58.1	57.9	59.0	59.0	59.3	60.6	60.9	61.0
	(SD)				(14.3)	(13.7)	(14.1)	(14.1)	(13.8)	(12.6)	(12.7)	(12.9)
	Male %	-	-	-	65	66	64	64	64	57	55	55

JMDC, Japan Medical Data Center; MDV, Medical Data Vision; SD, standard deviation.

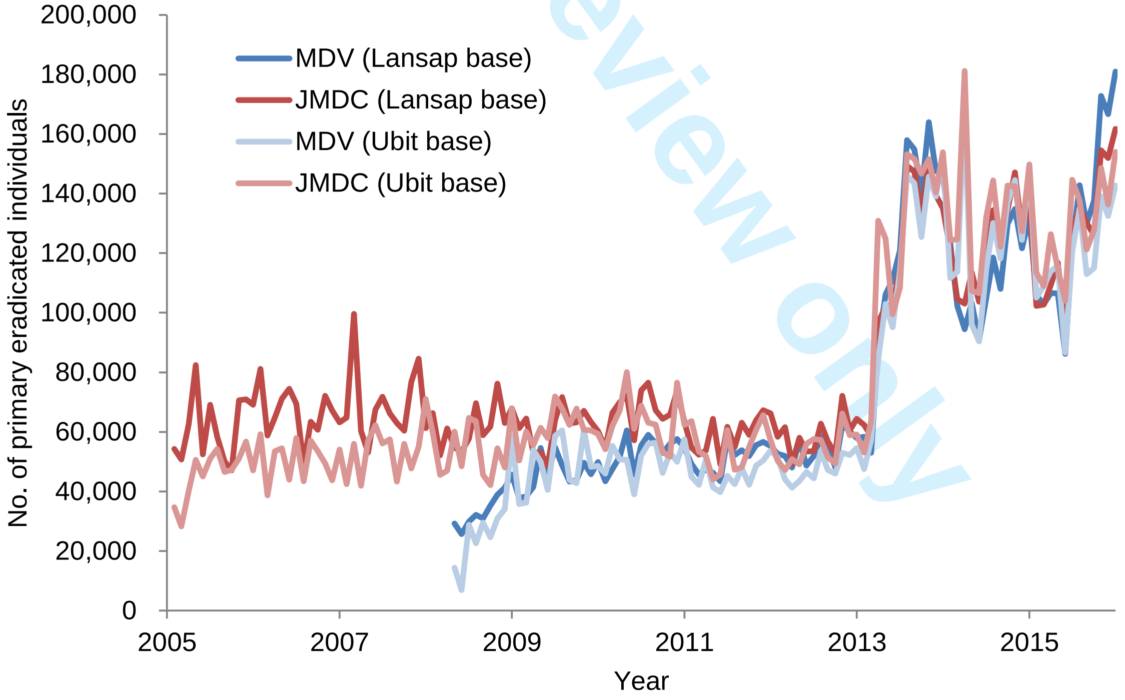

Figure S1. The number of individuals with the primary eradication of *H. pylori* according to each database.

The RECORD statement – checklist of items, extended from the STROBE statement, that should be reported in observational studies using routinely collected health data.

	Item No.	STROBE items	Location in manuscript where items are reported	RECORD items	Location in manuscript where items are reported
**Title and abstract**					
	1	(a) Indicate the study's design with a commonly used term in the title or the abstract (b) Provide in the abstract an informative and balanced summary of what was done and what was found	Title and Abstract; page 1–2.	RECORD 1.1: The type of data used should be specified in the title or abstract. When possible, the name of the databases used should be included.<br><br>RECORD 1.2: If applicable, the geographic region and timeframe within which the study took place should be reported in the title or abstract.<br><br>RECORD 1.3: If linkage between databases was conducted for the study, this should be clearly stated in the title or abstract.	Mentioned the type of data in Title and Abstract; page 1–2.<br><br>Reported the region in Title and Abstract; page 1–2.<br><br>N/A
**Introduction**					
Background rationale	2	Explain the scientific background and rationale for the investigation being reported	The 1st, 2nd and 3rd paragraphs of Introduction; page 5–7.		
Objectives	3	State specific objectives, including any prespecified hypotheses	The 4th paragraph (last paragraph) of Introduction; page 7.		
**Methods**					
Study Design	4	Present key elements of study design early in the paper	Methods (Study design section); page 9.		
Setting	5	Describe the setting, locations, and relevant dates, including periods of recruitment, exposure, follow-up, and data collection	Methods (Data sources section) ; page 7–8.		

For peer review only

Participants	6	*(a) Cohort study* - Give the eligibility criteria, and the sources and methods of selection of participants. Describe methods of follow-up<br>*Case-control study* - Give the eligibility criteria, and the sources and methods of case ascertainment and control selection. Give the rationale for the choice of cases and controls<br>*Cross-sectional study* - Give the eligibility criteria, and the sources and methods of selection of participants<br><br>*(b) Cohort study* - For matched studies, give matching criteria and number of exposed and unexposed<br>*Case-control study* - For matched studies, give matching criteria and the number of controls per case		RECORD 6.1: The methods of study population selection (such as codes or algorithms used to identify subjects) should be listed in detail. If this is not possible, an explanation should be provided.<br><br>RECORD 6.2: Any validation studies of the codes or algorithms used to select the population should be referenced. If validation was conducted for this study and not published elsewhere, detailed methods and results should be provided.<br><br>RECORD 6.3: If the study involved linkage of databases, consider use of a flow diagram or other graphical display to demonstrate the data linkage process, including the number of individuals with linked data at each stage.	The 1st paragraph of Patient identification and statistical analysis section in Methods; page 9–10.<br><br>Provided the detailed methods and results in the 1st paragraph of Patient identification and statistical analysis section in Methods and Patient characteristics section in Results page 9–11, 14.<br><br>N/A
Variables	7	Clearly define all outcomes, exposures, predictors, potential confounders, and effect modifiers. Give diagnostic criteria, if applicable.	Methods (Patient identification and statistical analysis section); page 10–13.	RECORD 7.1: A complete list of codes and algorithms used to classify exposures, outcomes, confounders, and effect modifiers should be provided. If these cannot be reported, an explanation should be provided.	Methods (Patient identification and statistical analysis section); page 10–13.
Data sources/ measurement	8	For each variable of interest, give sources of data and details of methods of assessment	Methods (Patient identification and statistical analysis		

		(measurement). Describe comparability of assessment methods if there is more than one group	section); page 9–11. We used two types of claims databases and two types of product sales data; therefore, four types of combination were obtained and then the combinations were compared (Figure S1)		
Bias	9	Describe any efforts to address potential sources of bias	Discussion (The 2nd paragraph of Strengths and limitations of this study section); page 18–19.		
Study size	10	Explain how the study size was arrived at	Methods (The 1st paragraph of Patient identification and statistical analysis section); page 9–10.		
Quantitative variables	11	Explain how quantitative variables were handled in the analyses. If applicable, describe which groupings were chosen, and why	Methods (Patient identification and statistical analysis section); page 11–13.		
Statistical methods	12	(a) Describe all statistical methods, including those used to control for confounding<br>(b) Describe any methods used to examine subgroups and interactions<br>(c) Explain how missing data were addressed<br>(d) *Cohort study* - If applicable, explain how loss to follow-up was addressed	Methods (Patient identification and statistical analysis section); page 10–13.		

For peer review only

		Case-control study - If applicable, explain how matching of cases and controls was addressed<br>*Cross-sectional study* - If applicable, describe analytical methods taking account of sampling strategy<br>(e) Describe any sensitivity analyses			
Data access and cleaning methods		..		RECORD 12.1: Authors should describe the extent to which the investigators had access to the database population used to create the study population.<br><br>RECORD 12.2: Authors should provide information on the data cleaning methods used in the study.	Methods (Data sources section); page 7.<br><br>N/A
Linkage		..		RECORD 12.3: State whether the study included person-level, institutional-level, or other data linkage across two or more databases. The methods of linkage and methods of linkage quality evaluation should be provided.	N/A
**Results**					
Participants	13	(a) Report the numbers of individuals at each stage of the study (*e.g.*, numbers potentially eligible, examined for eligibility, confirmed eligible, included in the study, completing follow-up, and analysed)<br>(b) Give reasons for non-participation at each stage.<br>(c) Consider use of a flow diagram	Results (Patient characteristics section); page 14.	RECORD 13.1: Describe in detail the selection of the persons included in the study (*i.e.,* study population selection) including filtering based on data quality, data availability and linkage. The selection of included persons can be described in the text and/or by means of the study flow diagram.	Results (Patient characteristics section); page 14.
Descriptive data	14	(a) Give characteristics of study	Results (Patient		

For peer review only

		participants (*e.g.*, demographic, clinical, social) and information on exposures and potential confounders<br>(b) Indicate the number of participants with missing data for each variable of interest<br>(c) *Cohort study* - summarise follow-up time (*e.g.*, average and total amount)	characteristics section and Table S1); page 14.		
Outcome data	15	*Cohort study* - Report numbers of outcome events or summary measures over time<br>*Case-control study* - Report numbers in each exposure category, or summary measures of exposure<br>*Cross-sectional study* - Report numbers of outcome events or summary measures	Results (Trend in the number of individuals with the primary eradication therapy and successful eradication section); page 14.		
Main results	16	(a) Give unadjusted estimates and, if applicable, confounder-adjusted estimates and their precision (e.g., 95% confidence interval). Make clear which confounders were adjusted for and why they were included<br>(b) Report category boundaries when continuous variables were categorized<br>(c) If relevant, consider translating estimates of relative risk into absolute risk for a meaningful time period	Results (Trend in the number of individuals with the primary eradication therapy and successful eradication section and Figure 1, and Trend and prediction of H. pylori infection and the effect of insurance policy changes section and Figure 3); page 14–15.		
Other analyses	17	Report other analyses done—e.g., analyses of subgroups and interactions, and sensitivity	N/A		

For peer review only

		analyses			
**Discussion**					
Key results	18	Summarise key results with reference to study objectives	The 1st paragraph of Discussion; page 15–16.		
Limitations	19	Discuss limitations of the study, taking into account sources of potential bias or imprecision. Discuss both direction and magnitude of any potential bias	Discussion (The 2nd paragraph of Strengths and limitations of this study section); page 18-19.	RECORD 19.1: Discuss the implications of using data that were not created or collected to answer the specific research question(s). Include discussion of misclassification bias, unmeasured confounding, missing data, and changing eligibility over time, as they pertain to the study being reported.	Discussion (The 2nd paragraph of Strengths and limitations of this study section); page 18-19.
Interpretation	20	Give a cautious overall interpretation of results considering objectives, limitations, multiplicity of analyses, results from similar studies, and other relevant evidence	The 2nd, 3rd and 4th paragraphs of Discussion; page 16-17.		
Generalisability	21	Discuss the generalisability (external validity) of the study results	Discussion (The 2nd and 3rd paragraphs of Strengths and limitations of this study section) ;page 18-19.		
**Other Information**					
Funding	22	Give the source of funding and the role of the funders for the present study and, if applicable, for the original study on which the present article is based	Footnote (Funding); page 20.		
Accessibility of protocol, raw data, and programming code				RECORD 22.1: Authors should provide information on how to access any supplemental information such as the study protocol, raw data, or programming code.	N/A

For peer review only

*Reference: Benchimol EI, Smeeth L, Guttmann A, Harron K, Moher D, Petersen I, Sørensen HT, von Elm E, Langan SM, the RECORD Working Committee. The REporting of studies Conducted using Observational Routinely-collected health Data (RECORD) Statement. *PLoS Medicine* 2015; in press.

*Checklist is protected under Creative Commons Attribution (CC BY) license.

For peer review only

BMJ Open

Impact of health insurance coverage for *Helicobacter pylori* gastritis on the trends in eradication therapy in Japan: retrospective observational study and simulation study based on real-world data.

Journal:	*BMJ Open*
Manuscript ID	bmjopen-2017-015855.R2
Article Type:	Research
Date Submitted by the Author:	23-May-2017
Complete List of Authors:	Hiroi, Shinzo ; Graduate School of Medicine and Public Health, Kyoto University, Department of Pharmacoepidemiology; Takeda Pharmaceutical Company, Limited, Japan Medical Affairs<br>Sugano, Kentaro; Jichi Medical University, Department of Medicine<br>Tanaka, Shiro; Graduate School of Medicine, Kyoto University, Department of Clinical Biostatistics<br>Kawakami, Koji; Graduate School of Medicine and Public Health, Kyoto University, Department of Pharmacoepidemiology
**Primary Subject Heading**:	Epidemiology
Secondary Subject Heading:	Gastroenterology and hepatology
Keywords:	health insurance, <i>Helicobacter pylori</i>, <i>Helicobacter pylori</i> eradication, <i>Helicobacter pylori</i>-positive gastritis

SCHOLARONE™
Manuscripts

For peer review only

Impact of health insurance coverage for *Helicobacter pylori* gastritis on the trends in eradication therapy in Japan: retrospective observational study and simulation study based on real-world data.

Shinzo Hiroi, HEOR Head[1,4], Kentaro Sugano, Professor Emeritus[2], Shiro Tanaka, Associate Professor[3], Koji Kawakami, Professor[1]

1 Department of Pharmacoepidemiology, Graduate School of Medicine and Public Health, Kyoto University, Kyoto, Japan

2 Department of Medicine, Jichi Medical University, Tochigi, Japan

3 Department of Clinical Biostatistics, Graduate School of Medicine, Kyoto University, Kyoto, Japan

4 Japan Medical Affairs, Takeda Pharmaceutical Company, Limited, Tokyo, Japan

Corresponding author:

Koji Kawakami

Department of Pharmacoepidemiology, Graduate School of Medicine and Public Health, Kyoto University, Yoshida Konoecho, Sakyoku, Kyoto 606-8501, Japan.

Tel: +81 75 753 9469

Fax: +81 75 753 4469

E-mail address: kawakami.koji.4e@kyoto-u.ac.jp

Keywords: health insurance, *Helicobacter pylori*, *Helicobacter pylori* eradication, *Helicobacter pylori*-positive gastritis

For peer review only - http://bmjopen.bmj.com/site/about/guidelines.xhtml

Abstract

Objectives

To explore the prevalence of *Helicobacter pylori (H. pylori)* infection in Japan and the trends of its eradication therapy before and after the changes of the insurance coverage policy, first started in 2000, and expanded to cover *H. pylori*-positive gastritis in 2013. The impacts the changes brought were estimated.

Methods

In this retrospective observational study and simulation study based on health insurance claims data, product sales data, and relevant studies, individuals who received triple therapy (amoxicillin, clarithromycin, proton-pump inhibitors or potassium-competitive acid blockers) were defined as the first-time patients for *H. pylori* eradication in two Japanese health insurance claims databases (from approximately 1.6 million and 10.5 million individuals). The each sales data of eradication packages and examination kits were used to estimate the number of *H. pylori*–eradicated individuals nationwide. The prevalence of *H. pylori* infection, including the future rate, was predicted using previous studies and the estimated population trend by a national institute. Cases completed prior to the policy change on insurance coverage were simulated to estimate what would have happened had there been no change in the policy.

Results

The numbers of patients first received eradication therapy were 81,119 and 170,993 from two databases. The nationwide estimated number of patients successfully eradicated was approximately 650,000 per year between 2001 and 2012, while it rapidly rose to 1,380,000 per year in 2013. The estimated prevalence of infection in 2050 is 5%; this rate was estimated to be 28% and 22% if the policy changes had not occurred in

For peer review only - http://bmjopen.bmj.com/site/about/guidelines.xhtml

2000 and 2013, respectively.

Conclusions

The impact of policy changes for *H. pylori* eradication therapy on the prevalence of infection was shown. The results suggest that insurance coverage expansion may also reduce the prevalence in other countries with a high prevalence of *H. pylori* infection if the reinfection is low.

For peer review only

For peer review only - http://bmjopen.bmj.com/site/about/guidelines.xhtml

Strengths and limitations of this study

- Demonstrates for the first time the impact of insurance policy expansion for *H. pylori* eradication therapy in a quantitative manner based on an analysis of nationwide real world data.
- Robust and reliable results were obtained from combinations of large-scale insurance claims databases and sales data of the most commonly used eradication treatments and test kits.
- The success rate of eradication was obtained from previous studies; therefore, the rate might be different from current clinical practice.
- The health insurance claims databases have potential biases: in one database, the information on individuals older than 65 years is limited because it is the information from employed individuals and their family members; another database included the data only from large hospitals.

For peer review only - http://bmjopen.bmj.com/site/about/guidelines.xhtml

Introduction

Throughout the world, gastric cancer is one of the most common cancers; 952,000 new patients were diagnosed in 2012.[1] The incidence of gastric cancer is higher in Asian countries; Korea, Japan, and China have the 1st, 3rd, and 5th highest rates, respectively, in the world.[2] In Japan, the prevalence and mortality of gastric cancer are constantly among the top three of all cancers. Therefore, it is considered to be one of the highest priorities in preventive policy. *Helicobacter pylori (H. pylori)* can cause gastric inflammation, which can then lead to gastric and duodenal ulcers, as well as gastric cancer.[3-5] Thus eradication of *H. pylori* is considered as an effective therapy in reducing the risk of those diseases.

Due to the concern of high gastric cancer prevalence in East Asian countries, some preventive programs have been launched to reduce the incidence of gastric cancer. In Korea, a cancer screening program was established by the government to provide for almost all people of eligible age (40 years or older for gastric cancer) with free screening or provision at minimum cost in 1999.[6] Large clinical trials and health economic studies have been conducted in China, and a consensus statement was formulated to encourage *H. pylori* eradication therapy.[6, 7] In Taiwan, the results of a community-level large screening and eradication program, as well as a health economic evaluation, support the efficacy of *H. pylori* eradication therapy.[8] In Japan, in November 2000, based on the results of diverse clinical studies,[3, 9-20] the government approved the addition of *H. pylori* eradication therapy in their insurance policy as a treatment for *H. pylori*-positive gastric ulcer and duodenal ulcer. Furthermore, insurance coverage was expanded in June 2010 to include gastric mucosa-associated lymphoid tissue lymphoma, idiopathic thrombocytopenic purpura, and post-endoscopic resection of early gastric

For peer review only - http://bmjopen.bmj.com/site/about/guidelines.xhtml

cancer, and in February 2013, to include *H. pylori*-positive gastritis based on the recommendations of Japanese guideline.[21]

Japanese health insurance is a system of universal coverage; the effect of change in health insurance coverage policy is spread throughout the nation. In terms of *H. pylori* eradication, everyone diagnosed as a disease covered for eradication therapy by health insurance can receive eradication therapy with coverage. Therefore, health insurance reimbursement seems to have the same or greater impact on clinical practice as recommendations from diagnostic/treatment guidelines in countries where universal health insurance coverage is established, such as in Japan and Korea. Various sizes of preventative programs for gastric cancer have been implemented in the high prevalence countries for both gastric cancer and *H. pylori* infection. In some countries, *H. pylori* eradication therapy for patients with *H. pylori*-positive gastric ulcer and duodenal ulcer has been covered by national health insurance. However, eradication therapy for *H. pylori*-positive gastritis has not been covered to date in these countries other than Japan.[8] The effect of insurance coverage expansion on the prevalence of *H. pylori* infection has been evaluated in only a few studies at the community level in Japan.[22,23] Nonetheless, the national-level prevalence rate of *H. pylori* infection has not been reported and its change has not been assessed after the insurance coverage for *H. pylori* eradication therapy was expanded to include *H. pylori*-positive gastritis in 2013.The progressive insurance expansion was reported to be efficient[24] and the incidence of peptic ulcer has decreased since the change in insurance coverage policy for *H. pylori* eradication in 2000.[25] They also estimated that gastric cancer mortality would decrease based on the assumption that 50% of *H. pylori*-infected patients would receive eradication therapy.[26] However, this estimate was based on neither the observed number

For peer review only - http://bmjopen.bmj.com/site/about/guidelines.xhtml

of patients undergoing *H. pylori* eradication nor the prevalence rate of infection. To evaluate the impact of changes to the insurance policy on the incidence of various diseases, including gastric cancer, it is necessary to elucidate the national trend of eradication therapy and the prevalence rate of infection before and after the changes in the insurance policy.

The primary objective of this study was to assess how health insurance policy changes have impacted eradication therapy and the prevalence rate of *H. pylori* infection in Japan. Furthermore, the future effect, as a result of the policy changes, on the prevalence of *H. pylori* infection was evaluated. In this study, health insurance claims databases and product sales data were used to estimate the number of eradication treatments. The successful eradication rate from 2000 onwards, at which time the health insurance began its coverage for the eradication therapy, was also estimated. The prevalence rate of infection and the number of infected individuals up to 2060 were predicted as based on the above data analysis and the prevalence rate of *H. pylori* infection as reported in previous studies. Furthermore, a simulation was conducted to estimate what the probable effects would be had the policy changes not been made.

Methods

Data sources

Insurance claims databases from Japan Medical Data Center (JMDC) from January 2005 to December 2015 and Medical Data Vision (MDV) from April 2008 to December 2015 were used for the analyses. The JMDC database is a registry of health insurance claims and medical examination records for insured individuals and their families in more than 50 health insurance societies. Because this database only included

For peer review only - http://bmjopen.bmj.com/site/about/guidelines.xhtml

information on company employees and their families, the information for those older than 65 years was limited. Also, there were no data for those older than 75 years. Until 2014, this database covered 1.6 million individuals which accounted for 1% of the Japanese population. The medical database from MDV covered 10.5 million individuals in 192 acute care hospitals using diagnostic procedure combination/per-diem payment system (DPC/PDPS). It included 11% of acute care hospitals in Japan with the number of beds from 20 to more than 1,000. These databases included the patient's gender, age, diagnosis, prescription information, and so on. Diagnosis information is based on the International Classification of Diseases 10th Revision, and drugs are coded in the Anatomical Therapeutic Chemical Classification System. Both databases included anonymous and personally unidentifiable data.

To estimate the nationwide number of infected individuals, product sales data for the most common eradication medicine, and test kit for *H. pylori* infection were analyzed. The sales data for eradication medicine, Lansap® (Takeda Pharmaceutical Co., Ltd.), which consists of lansoprazole, amoxicillin, and clarithromycin in one package was provided by the manufacturer from December 2002 through December 2015. The data for ^{13}Urea Breath Test (UBIT®, Otsuka Pharmaceutical Co., Ltd.) was obtained as well from November 2000 through December 2015.

To determine the trend in the number of *H. pylori* infected individuals, previously published Japanese studies were used (Table 1).

Table 1. Studies on the number of *H. pylori* infected individuals and prevalence rate of infection

First Author	Number of Subjects	Population	Study Design	Observation Year
Asaka M[27]	426	Asymptomatic children, students and adults (participating at the health screening center) living in Sapporo, Hokkaido	Observational study	1990

For peer review only - http://bmjopen.bmj.com/site/about/guidelines.xhtml

Fujisawa T[28]	349 (1974)<br>324 (1984)<br>342 (1994)	Healthy persons living in 7 prefectures in the central part of Japan	Observational study	1974<br>1984<br>1994
Watabe H[29]	6,983	Participants in a mass health appraisal programme.	Observational study	1996
Ueda J[30]	14,716	Individuals who underwent a health checkup in 7 prefectures (Hokkaido, Aomori, Yamagata, Gunma, Aichi, Shiga and Kagawa)	Observational study	2005
Shiota S[31]	5,550	Patients of Oita University Hospital, Oita, Japan	Observational study	2009

Study design

This study is a retrospective observational study and simulation study based on the health insurance claims data, product sales data, and relevant published studies. The steps were as follows; firstly, the number of individuals who received the eradication therapy and those who had successful eradication were estimated based on the analyses of the health insurance claims databases and product sales data. Secondly, the trend in the number of *H. pylori* infected individuals was determined from previously published studies. Thirdly, the prevalence rate and trend of *H. pylori* infection was estimated and forecasted from the results of the first and second step. Finally, to fully evaluate the impact of the policy changes, a simulation was made considering effects which likely would have occurred without the insurance policy changes in 2000 and 2013.

Patient identification and analysis

In the JMDC and MDV databases, the individuals who received triple therapy, either the primary eradication package (such as Lansap® and other packaged products), or the combination of amoxicillin, clarithromycin, and either proton-pump inhibitors or potassium-competitive acid blockers (all prescribed within the same month), were defined as individuals with primary eradication of *H. pylori*. The drugs used for the

For peer review only - http://bmjopen.bmj.com/site/about/guidelines.xhtml

therapy were defined by product name in JMDC database and remuneration code in MDV database. The examination was defined by remuneration code, and the diagnosis for those who had eradicated was defined by name of diagnosis in both databases. To estimate the number of individuals who received primary eradication therapy in the nation, the following were calculated in these databases:

1) Percentage of individuals who used Lansap® for primary eradication therapy to all individuals who received the primary eradication.
2) Percentage of individuals who received UBIT® for the *H. pylori* test after eradication to all individuals who took the *H. pylori* test.

To calculate the number of individuals who achieved successful eradication, the following were assumed (Figure 1):

- The primary and secondary success rates of eradication for this study were presumed to be 75% and 90%, respectively, based on previous studies in Japan.[25, 27] Secondary eradication was premised to be performed for all those who failed primary eradication. Therefore, the success rate of the eradications was estimated to be 98% of the primary eradication, and the percentage was used as the success rate in this study.
- New infection in adulthood was reported to be rare[32] and reinfection per year after the eradication therapy in Japan is reported to be approximately 1%;[33,34] however it was assumed to be 0% in this study.

The nationwide number of individuals who received primary eradication was estimated based on 1) and 2) with sales data of Lansap® and UBIT® as follows:

- The monthly number of individuals who received primary eradication from January 2010 was calculated as the mean of four estimates (Lansap®-base from

For peer review only - http://bmjopen.bmj.com/site/about/guidelines.xhtml

MDV and JMDV, UBIT®-base from MDV and JMDC).

- The monthly number of individuals who received primary eradication from January 2006 to December 2009 was calculated as the mean of two estimates from the JMDC database (Lansap®-base and UBIT®-base).
- The monthly number of individuals who received primary eradication from November 2000 to December 2005 was extrapolated using the sales number of UBIT® in each month and the UBIT® share rate in 2006 on the assumption that the share rate in this period was the same as that in 2006.

The formula used is as follows:

$(\text{Successful Eradication Number})_{YM}$ = $(\text{Primary Successful Eradication Number})_{YM}$ + $(\text{Secondary Successful Eradication Number})_{YM}$

$(\text{Primary Successful Eradication Number})_{YM}$ = $(\text{Primary Eradication Number})_{YM}$ × (Primary Eradication Success Rate, 75%)

$(\text{Secondary Successful Eradication Number})_{YM}$ = [$(\text{Primary Eradication Number})_{YM}$ - $(\text{Primary Successful Eradication Number})_{YM}$] × (Secondary Eradication Success Rate, 90%)

$(\text{Primary Eradication Number})_{YM}$ = [$(\text{Primary Eradication Number with Lansap®})_{YM,JMDC}$ + $(\text{Primary Eradication Number with Lansap®})_{YM,MDV}$ + $(\text{Primary Eradication Number with UBIT®})_{YM,JMDC}$ + $(\text{Primary Eradication Number with UBIT®})_{YM,MDV}$] / 4

$(\text{Primary Eradication Number with Lansap®})_{YM,Database}$ = $(\text{Sales Number of Lansap®})_{YM}$ / $(\text{Share of Lansap®})_{YM,Database}$

$(\text{Primary Eradication Number with UBIT®})_{YM,Database}$ = $(\text{Examination Number})_{YM,Database}$ × $(\text{Ratio of Primary Eradication to Total Examination})_{YM,Database}$

For peer review only - http://bmjopen.bmj.com/site/about/guidelines.xhtml

(Examination Number)$_{YM,Database}$ = (Sales Number of UBIT®)$_{YM}$ / (Share of UBIT®)$_{YM,Database}$

where YM is year month, and Database = JMDC or MDV database.

The number of infected individuals and prevalence rate of infection were estimated based on the previous Japanese studies shown in Table 1. The number of infected individuals until March 2013 was estimated from previous studies[27-31] and vital statistics in Japan conducted by Ministry of Health, Labour and Welfare.[35] An exponential decay approximation curve was calculated based on the results of the previous studies until 2000. After 2000, the estimated mean monthly number of individuals who achieved successful eradication from January 2001 to March 2013 was taken into account. The number of infected individuals from April 2013 was estimated using the estimated mean monthly number of individuals who achieved successful eradication from April 2013 to December 2015, giving consideration to the decrease in the number of infected individuals due to death. It was calculated as follows:

$$(\text{Infection Number})_{CY} = \frac{\sum_{DOBYR} \text{National Population}_{DOBYR,\ CY} \times \text{Prevalence Rate}_{DOBYR,\ CY}}{\sum_{DOBYR} \text{National Population}_{DOBYR,\ CY}}$$

where (Prevalence Rate)$_{DOBYR,CY}$ is the prevalence rate of *H. pylori* infection by birth year in each observation year calculated in this study, and (National Population)$_{DOBYR,CY}$ is the population by birth year in each observation year; where DOBYR is birth year by 5 years, and CY is the calendar year of observation of each study. An exponential parameter that minimizes the sum of squared distances from (Infection Number)$_{CY}$ was calculated (least squares method), assuming the decrease of the number of *H. pylori* infected individuals by any reason other than eradication (*i.e.,* aging) followed an exponential function and the decrease of that by eradication was constant from 2001

For peer review only - http://bmjopen.bmj.com/site/about/guidelines.xhtml

through 2013.

Assuming that *H. pylori* infection would be decreasing exponentially after 2015 (by consideration of the natural decrease due to the death of older infected individuals), a simulation was performed using the number of infected individuals obtained in the previous section and the population forecast from the National Institute of Population and Social Security Research[36] to predict the number of infected individuals in the future. The prevalence rate of infection was also simulated for the case of no policy change regarding insurance coverage in 2000 and 2013. This was simulated as follows:

1) The number of *H. pylori* infected individuals calculated in the previous section was taken to be $(\text{Infection Number})_{CY}$ before 2013.
2) The number of *H. pylori* infected individuals from 2013 through 2015 was assumed to be decreased from $(\text{Infection Number})_{CY}$ before 2013, based on the estimated number of individuals who achieved successful eradication by the analysis of the JMDC and MDV databases and sales data of drugs.
3) If Case 0, $(\text{Prevalence Rate})_{CY,case} = (\text{Infection Number})_{CY} / (\text{National Population})_{CY}$, in $1975 \leq CY \leq 2000$; or $(\text{Prevalence Rate})_{CY,case} / (\text{Prevalence Rate})_{CY-1,case} = (\text{Infection Number})_{CY-1} / (\text{Infection Number})_{CY-2}$, in $2016 \leq CY$.
4) If Case 1, $(\text{Prevalence Rate})_{CY,case} = (\text{Infection Number})_{CY} / (\text{National Population})_{CY}$, in $1975 \leq CY \leq 2000$;or $(\text{Prevalence Rate})_{CY,case} / (\text{Prevalence Rate})_{CY-1,case} = (\text{Infection Number})_{CY-1} / (\text{Infection Number})_{CY-2}$ in $2001 \leq CY$.
5) If Case 2, $(\text{Prevalence Rate})_{CY,case} = (\text{Infection Number})_{CY} / (\text{National Population})_{CY}$, in $1975 \leq CY \leq 2012$;or $(\text{Prevalence Rate})_{CY,case} / (\text{Prevalence Rate})_{CY-1,case} = (\text{Infection Number})_{CY-1} / (\text{Infection Number})_{CY-2}$ in $2013 \leq CY$.

For peer review only - http://bmjopen.bmj.com/site/about/guidelines.xhtml

Case 0 is with the current policy; Case 1 or 2 represent the case in which policy change had not occurred in 2000 or 2013.

Statistical analysis was carried out using Excel 2010 (Microsoft, Redmond, WA, USA) and SAS version 9.4 (SAS Institute, Cary, NC, USA).

Results

Patient characteristics

The total number of individuals who received primary eradication was 81,119 (mean age 36.8 years; males 61%) from the JMDC database, and 170,993 (mean age 60.6 years; males 57%) from the MDV database. The characteristics for each year are shown in Table S1 (see online supplementary appendix).

Trend in the number of individuals with the primary eradication therapy and successful eradication

The difference among the four (Lansap®-base from MDV and JMDV, UBIT®-base from MDV and JMDC) estimated numbers of individuals who received primary eradication was confirmed to be minimal after 2010 (see online supplementary appendix. Figure S1). The nationwide number of individuals who had successful eradication (both first-line and second-line) was estimated as shown in Figure 2. The number was approximately 650,000 per year between 2001 and 2012, which has reached a steady state of approximately 700,000 per year after an increase in 2006. However, there was a slight decrease in 2011. It markedly increased to 1,380,000 in 2013, which is more than double the number observed in 2012. In the diagnoses for those who had successful eradication treatment (Figure 2), gastritis accounted for more than half of the diagnoses

For peer review only - http://bmjopen.bmj.com/site/about/guidelines.xhtml

since 2013. The average number of individuals who received successful eradication treatment up to March 2013 was 54,000 per month, while it was 124,000 per month after March 2013. The cumulative total of individuals who received successful eradication treatment was more than 10 million up to September 2014.

Trend in the number of infected individuals and the prevalence rate of infection

Figure 3a illustrates the prevalence rate of *H. pylori* infection by birth year from previous studies. This shows higher prevalence rates of infection in the cohorts with earlier birth years. Also, there was a tendency for the difference of prevalence rates of infection among studies to be larger in those with an earlier birth year, and the rate was lower in later observations. The overall estimated prevalence rate of infection was lower in later years (Figure 3b). The lines were fitted after taking the effect of insurance policy change in November 2011 into account, shown in Figure 3b.

Trend and prediction of H. pylori infection and the effect of insurance policy changes

The pattern of the number of infected individuals from 2016 in Japan was predicted (blue broken line in Figure 4) based on the trend in the number of infected individuals derived from the results above and the population forecast.[35] The patterns, in the case without policy changes in insurance coverage in 2000 and 2013, were also simulated (red and green broken lines in Figure 4). The simulation showed that the number of infected individuals would decrease to 16,200,000 individuals in 2030, or 14% of the population, and further decrease to 5% of the population in 2050. These figures would have been 28% and 21% in 2030 if the policy changes had not occurred in 2000 and 2013, respectively.

For peer review only - http://bmjopen.bmj.com/site/about/guidelines.xhtml

For peer review only

Discussion

This study described the status of eradication therapy and trend of *H. pylori* infection using large insurance claims databases that reflected actual clinical practice at the national level in Japan. The analysis showed that the prevalence rate of *H. pylori* infection has decreased after the approval to include eradication therapy in the insurance policy in 2000. The number of successful eradications more than doubled immediately after insurance coverage for *H. pylori* eradication therapy was expanded in 2013. The simulation indicated that the prevalence rate of *H. pylori* infection would decrease and reach approximately 14% in 2030 and 5.4% in 2050.

Although it is difficult to compare the prevalence rate of infection among studies due to time and sample difference, it is worthwhile to compare the rate with other countries. The prevalence rate of *H. pylori* infection varies markedly in different countries; in general, it is higher in developing countries and lower in developed countries.[37] The prevalence rate of infection was reported to be 92% in Bangladesh,[38] 75% in Vietnam,[39] 41-72% in China,[40] and 54-60% in Korea,[41,42] while it was 15-22% in Australia[43,44] and 8-27% in the USA.[45,46] Although the estimate of the prevalence rate of *H. pylori* infection in our study was 43% in Japan in 2000, based on our simulation, the prevalence rate of infection in Japan in 2030 with expanded insurance coverage would be almost the same as the Australian rate (15%), and it would reach the North American level (8-27%) by approximately 2050.

The estimate from the previous studies indicated a higher prevalence rate of infection in older cohorts, which can be explained by environmental factors such as poor sanitation.[47,48] It has also been suggested that nowadays *H. pylori* infection occurs

For peer review only - http://bmjopen.bmj.com/site/about/guidelines.xhtml

in childhood in Japan.[49,50] As a result, the number of infections would have naturally decreased even without eradication therapy due to the death of older infected individuals. However, our simulation showed that the prevalence rate of infection would have been higher than 14% and 7% in 2030 if the policy changes had not occurred in 2000 and 2013 respectively. It was therefore evident that those insurance policy changes had contributed to the reduction in the prevalence of *H. pylori* infection. Japan has established a universal health insurance coverage system, which means that by law all Japanese residents are entitled to health insurance coverage for medical treatments. This system has a great impact on the dissemination of medical treatments; consequently the use of eradication therapy is highly influenced by the presence of the health insurance and coverage for diseases.

The results of this study could be a good indicator for the implementation of insurance coverage for eradication of *H. pylori* in countries where *H. pylori* is prevalent, especially the East Asian countries. Countries and regions, such as Korea, China, and Taiwan, have been conducting clinical trials of *H. pylori* eradication; however, they have not yet established any policy for *H. pylori* eradication. This study is likely to be used as one of the references in considering effect on policy change in such countries. This study might also be important in providing direction for future research in Japan. In 2016, the revised edition of the Japanese Guideline for Diagnosis and Treatment for *H. pylori* infection was published following that of 2009. In the latest guideline, the expansion of insurance coverage for the treatment for *H. pylori* gastritis in 2013 is described.[51] Also, a regimen with potassium-competitive acid blocker (P-CAB)-based triple therapy, which is newly described in the guideline, demonstrated a high eradication rate compared to the conventional proton pump inhibitor-based triple

For peer review only - http://bmjopen.bmj.com/site/about/guidelines.xhtml

therapy.[51,52] It seems that the prevalence rate of infection could be further reduced in Japan.

Comparison with other studies

There have been some community level studies investigating the status of eradication in clinical practices in Japan.[22, 23] Nevertheless, to our knowledge, there was no study describing the status of eradication therapy at the national level. Asaka *et al.* have reported that the insurance policy change could increase the number of eradications, reduce the number of infected individuals, and decrease mortality from gastric cancer.[24,25] They estimated that the number of deaths from gastric cancer would reach 60,000 in 2020 without any countermeasures, while it would be half if 50% of infected individuals receive eradication therapy. However, this assumption was not based on actual observations. Our study, using real world data reflecting actual medical practice, showed a rapid increase in patients receiving *H. pylori* eradication, after the insurance policy change in 2013.

Strengths and limitations of this study

Here, a national level evaluation using real world data, allowed us to analyze the impact of insurance policy expansion for *H. pylori* eradication therapy in a quantitative manner. Furthermore, robust and reliable results were obtained from combinations of large-scale insurance claims databases and sales data of the most commonly used eradication treatments and test kits.

However, this study has several limitations. First, the success rate of eradication was obtained from previous studies;[25,27] thus, the rate might be different

For peer review only - http://bmjopen.bmj.com/site/about/guidelines.xhtml

For peer review only

from clinical practice, including the possibility that it may be estimated higher than the actual rate considering the effect of the increase in bacteria resistant to antibiotics. Nevertheless, the success rate in this study is believed to be close to the actual rate. Second, the health insurance claims databases have potential biases. The information available was limited for those older than 65 years in the JMDC database because it consisted of information on employed individuals and their family members. In addition, those who were self-employed and employees in small-to-medium-sized enterprises were not included. The information in the MDV database was obtained from the hospital using DPC/PDPS. Indeed, the mean ages of individuals who received primary eradication in both databases were different. However, the product share rates calculated in both databases were very similar after 2010, and the estimates of the number of individuals with the primary eradication were almost identical in both databases. Therefore, the impact of the different age distribution was believed to be minimal, and the estimates are believed to be accurate.

Despite these limitations, this study used the information from reliable clinical studies and large databases covering a significant number of Japanese citizens. Consequently, it is believed that the information presented here reflects the clinical status in Japan.

Conclusion and policy implications

This study described and forecasted the trend of *H. pylori* eradication therapy and assessed the impact of insurance policy change on the prevalence rate of *H. pylori* infection. It has demonstrated that the policy change was associated with a reduction in the prevalence rate of *H. pylori* infection in Japan. Furthermore it is expected to lead to

For peer review only - http://bmjopen.bmj.com/site/about/guidelines.xhtml

For peer review only

a reduction in the incidence of gastric cancer. Adaption of a similar nationwide health insurance coverage plan for *H. pylori* eradication by other high risk countries and regions may reduce the prevalence of *H. pylori* infection in the short and medium terms and may also have the possibility to have a positive effect on the incidence of *H. pylori*-related conditions, including gastric cancer, in the future.

For peer review only

For peer review only - http://bmjopen.bmj.com/site/about/guidelines.xhtml

Acknowledgements

The authors thank Takeda's collaborator, Otsuka Pharmaceutical Co., Ltd. for providing sales data of urea breath test diagnostic kits (UBIT®). The authors thank Kosuke Iwasaki and Tomomi Takeshima, employees of Milliman Inc., for analyzing the database, and writing and editorial suppport, respectively, funded by Takeda Pharmaceutical Co., Ltd.

Contributors

SH, KS and KK contributed to the concept and design of the study.

SH contributed to acquisition of data.

SH contributed to the analysis.

SH, KS, and ST contributed to interpretation of data.

SH, KS, ST and KK contributed to the writing of the manuscript and critical revision of the manuscript.

All authors approved the final version of the manuscript.

KK is the guarantor of the article.

Funding

This study was sponsored by Takeda Pharmaceutical Co., Ltd.

Competing interests

SH is an employee of Takeda Pharmaceutical Co., Ltd.

KS has received research grants from Eisai, Daiichi Sankyo Pharm, and Takeda Pharmaceutical Co., Ltd. He has also received lecture fees from Astellas Pharma,

For peer review only - http://bmjopen.bmj.com/site/about/guidelines.xhtml

Fujifilm, and Takeda Pharmaceutical Co., Ltd.

ST has no personal interests to declare.

KK received research funds from Dainippon Sumitomo Pharma, Olympus, Stella Pharma, Medical Platform Co., Novartis Pharmaceutical K.K., Bayer, and Maruho; honorarium from Astellas, Daiichi Sankyo Pharm, Taisho Pharmaceutical, Eisai, Novartis Pharmaceutical K.K., Mitsubishi Tanabe Pharma, Takeda Pharmaceutical Co., Ltd, Sanofi K.K.; consult fees from Olympus, Kyowa Hakko Kirin, Kaken Pharmaceutical, and Otsuka Pharmaceutical Co., Ltd.

There are no patents, products in development or marketed products to declare, relevant to those companies.

Ethical approval

This study was approved by Ethic Committee, Kyoto University Graduate School and Faculty of Medicine (R0126-1). This study was exempted from obtaining individual informed consent based on Ethical Guidelines for Medical and Health Research Involving Human Subjects by Ministry of Education, Culture, Sports, Science and Technology, and Ministry of Health, Labour, and Welfare.

Provenance and peer review

Not commissioned; externally peer reviewed.

Data sharing statement

No additional data are available.

For peer review only

Transparency

The lead author (KK) affirms that the manuscript is an honest, accurate, and transparent account of the study reported; that no important aspects of the study have been omitted; and that any discrepancies from the study as planned have been explained.

This is an Open Access article distributed in accordance with the Creative Commons Attribution Non Commercial (CC BY-NC 3.0) license, which permits others to distribute, remix, adapt, build upon this work non-commercially, and license their derivative works on different terms, provided the original work is properly cited and the use is non-commercial. See: http://creativecommons.org/licenses/by-nc/3.0/.

For peer review only

References

1. Ferlay J, Soerjomataram I, Dikshit R, et al. Cancer incidence and mortality worldwide: sources, methods and major patterns in GLOBOCAN 2012. *Int J Cancer* 2015;136:E359-86 doi: 10.1002/ijc.29210.

2. World Cancer Research Fund International. Stomach cancer statistics. 2016. http://www.wcrf.org/int/cancer-facts-figures/data-specific-cancers/stomach-cancer-statistics.

3. Fukase K, Kato M, Kikuchi S, et al. Effect of eradication of *Helicobacter pylori* on incidence of metachronous gastric carcinoma after endoscopic resection of early gastric cancer: an open-label, randomised controlled trial. *Lancet* 2008;372:392-97 doi: 10.1016/S0140-6736(08)61159-9.

4. Marshall BJ, Armstrong JA, McGechie DB, et al. Attempt to fulfil Koch's postulates for pyloric *Campylobacter*. *Med J Aust* 1985;142:436-39.

5. Morris A, Nicholson G. Ingestion of *Campylobacter pyloridis* causes gastritis and raised fasting gastric pH. *Am J Gastroenterol* 1987;82:192-99.

6. IARC Working Group. *Helicobacter pylori* eradication as a strategy for preventing gastric cancer. 2014. https://www.iarc.fr/en/publications/pdfs-online/wrk/wrk8/Helicobacter_pylori_Eradication.pdf.

7. Liu WZ, Xie Y, Cheng H, et al. Fourth Chinese National Consensus Report on the management of *Helicobacter pylori* infection. *J Dig Dis* 2013;14:211-21 doi: 10.1111/1751-2980.12034.

8. Lee S-Y. Current progress toward eradicating *Helicobacter pylori* in East Asian countries: differences in the 2013 revised guidelines between China, Japan, and

For peer review only - http://bmjopen.bmj.com/site/about/guidelines.xhtml

South Korea. *World J Gastroenterol* 2014;20:1493-502 doi: 10.3748/wjg.v20.i6.1493.

9. Asaka M, Kato M, Sugiyama T, et al. Follow-up survey of a large-scale multicenter, double-blind study of triple therapy with lansoprazole, amoxicillin, and clarithromycin for eradication of *Helicobacter pylori* in Japanese peptic ulcer patients. *J Gastroenterol* 2003;38:339-47 doi: 10.1007/s005350300061.

10. Emilia G, Longo G, Luppi M, et al. *Helicobacter pylori* eradication can induce platelet recovery in idiopathic thrombocytopenic purpura. *Blood* 2001;97:812-14 doi: 10.1182/blood.V97.3.812.

11. Gasbarrini A, Franceschi F, Tartaglione R, et al. Regression of autoimmune thrombocytopenia after eradication of *Helicobacter pylori*. *Lancet* 1998;352:878-78 doi: 10.1016/S0140-6736(05)60004-9.

12. Hentschel E, Brandstatter G, Dragosics B, et al. Effect of ranitidine and amoxicillin plus metronidazole on the eradication of *Helicobacter pylori* and the recurrence of duodenal ulcer. *N Engl J Med* 1993;328:308-12 doi: 10.1056/NEJM199302043280503.

13. Leodolter A, Kulig M, Brasch H, et al. A meta‐analysis comparing eradication, healing and relapse rates in patients with *Helicobacter pylori*‐associated gastric or duodenal ulcer. *Aliment Pharmacol Ther* 2001;15:1949-58 doi: 10.1046/j.1365-2036.2001.01109.x.

14. Malfertheiner P, Megraud F, O'Morain C, et al. Current concepts in the management of *Helicobacter pylori* infection: the Maastricht III Consensus Report. *Gut* 2007;56:772-81 doi: 10.1136/gut.2006.101634.

15. Miwa H, Sakaki N, Sugano K, et al. Recurrent peptic ulcers in patients following

For peer review only - http://bmjopen.bmj.com/site/about/guidelines.xhtml

successful *Helicobacter pylori* eradication: a multicenter study of 4940 patients. *Helicobacter* 2004;9:9-16 doi: 10.1111/j.1083-4389.2004.00194.x.

16. Ogura K, Hirata Y, Yanai A, et al. The effect of *Helicobacter pylori* eradication on reducing the incidence of gastric cancer. *J Clin Gastroenterol* 2008;42:279-83 doi: 10.1097/01.mcg.0000248006.80699.7f.

17. Take S, Mizuno M, Ishiki K, et al. The effect of eradicating *Helicobacter pylori* on the development of gastric cancer in patients with peptic ulcer disease. *Am J Gastroenterol* 2005;100:1037-42 doi: 10.1111/j.1572-0241.2005.41384.x.

18. Takenaka R, Okada H, Kato J, et al. *Helicobacter pylori* eradication reduced the incidence of gastric cancer, especially of the intestinal type. *Aliment Pharmacol Ther* 2007;25:805-12 doi: 10.1111/j.1365-2036.2007.03268.x.

19. Wong BC-Y, Lam SK, Wong WM, et al. *Helicobacter pylori* eradication to prevent gastric cancer in a high-risk region of China: a randomized controlled trial. *JAMA* 2004;291:187-94 doi: 10.1001/jama.291.2.187.

20. Wotherspoon AC, Diss TC, Pan L, et al. Regression of primary low-grade B-cell gastric lymphoma of mucosa-associated lymphoid tissue type after eradication of *Helicobacter pylori*. *Lancet* 1993;342:575-77 doi: 10.1016/0140-6736(93)91409-F.

21. Asaka M, Kato M, Takahashi S, et al. Guidelines for the management of *Helicobacter pylori* infection in Japan: 2009 revised edition. *Helicobacter.* 2010;15:1-20 doi: 10.1111/j.1523-5378.2009.00738.x.

22. Hanamure Y, Honda A, Hokonohara A. [Eradication therapy in the era of *Helicobacter pylori* infection -Its issues-: Current status and issues of *Helicobacter pylori* treatment in Ichikikushikino-city (Kagoshima prefecture)]. *Helicobacter Research* 2014;18:74-77. Japanese.

For peer review only - http://bmjopen.bmj.com/site/about/guidelines.xhtml

23. Fujimori K. [The changes in *Helicobacter pylori* infection treatment: Is the number of infection reduced?]. *Helicobacter Research* 2014;18:314-319. Japanese.

24. Asaka M, Kato M, Sakamoto N. Roadmap to eliminate gastric cancer with *Helicobacter pylori* eradication and consecutive surveillance in Japan. *J Gastroenterol* 2014;49:1-8 doi: 10.1007/s00535-013-0897-8.

25. Sugano K, Osawa H, Satoh K. Clinical Management of *Helicobacter pylori*-The Japanese Perspective. *Dig Dis* 2014;32:281-89 doi: 10.1159/000357859.

26. Asaka M, Mabe K, Matsushima R, et al. *Helicobacter pylori* eradication to eliminate gastric cancer: the Japanese strategy. *Gastroenterol Clin North Am* 2015;44:639-48 doi: 10.1016/j.gtc.2015.05.010.

27. Asaka M, Kimura T, Kudo M, et al. Relationship of *Helicobacter pylori* to serum pepsinogens in an asymptomatic Japanese population. *Gastroenterology* 1992;102:760-66 doi: 10.1016/0016-5085(92)90156-S.

28. Fujisawa T, Kumagai T, Akamatsu T, et al. Changes in seroepidemiological pattern of *Helicobacter pylori* and hepatitis A virus over the last 20 years in Japan. Am J Gastroenterol 1999;94:2094-99 doi: 10.1111/j.1572-0241.1999.01283.x.

29. Watabe H, Mitsushima T, Yamaji Y, et al. Predicting the development of gastric cancer from combining *Helicobacter pylori* antibodies and serum pepsinogen status: a prospective endoscopic cohort study. Gut 2005;54:764-68 doi: 10.1136/gut.2004.055400.

30. Ueda J, Gosho M, Inui Y, et al. Prevalence of *Helicobacter pylori* infection by birth year and geographic area in Japan. Helicobacter 2014;19:105-10 doi: 10.1111/hel.12110.

31. Shiota S, Murakawi K, Suzuki R, et al. *Helicobacter pylori* infection in Japan.

For peer review only - http://bmjopen.bmj.com/site/about/guidelines.xhtml

Expert Rev Gastroenterol Hepatol 2013;7:35-40 doi: 10.1586/egh.12.67.

32. Cave DR. How is *Helicobacter pylori* transmitted? *Gastroenterology* 1997;113:S9-14 doi: 10.1016/S0016-5085(97)80004-2.

33. Take S, Mizuno M, Ishiki K, et al. Reinfection rate of *Helicobacter pylori* after eradication treatment: a long-term prospective study in Japan. *J Gastroenterol* 2012;47:641-6 doi: 10.1007/s00535-012-0536-9.

34. Okimoto T, Murakami K, Sato R, et al. Is the recurrence of *Helicobacter pylori* infection after eradication therapy resultant from recrudescence or reinfection, in Japan. *Helicobacter* 2003;8:186-91 doi: 10.1046/j.1523-5378.2003.00143.x.

35. Ministry of Health, Labour and Welfare. [Summary Report of Vital Statistics of Japan 2013 (final data)]. 2014. http://www.mhlw.go.jp/toukei/saikin/hw/jinkou/kakutei13/index.html. Japanese.

36. National Institute of Population and Social Security Research. [Future Population Prediction in Japan]. 2012. http://www.ipss.go.jp/syoushika/tohkei/newest04/sh2401top.html. Japanese.

37. Fock KM, Ang TL. Epidemiology of *Helicobacter pylori* infection and gastric cancer in Asia. *J Gastroenterol Hepatol* 2010;25:479-86 doi: 10.1111/j.1440-1746.2009.06188.x.

38. Ahmad MM, Rahman M, Rumi AK, et al. Prevalence of *Helicobacter pylori* in asymptomatic population--a pilot serological study in Bangladesh. *J Epidemiol* 1997;7:251-54 doi: 10.2188/jea.7.251.

39. Hoang TTH, Bengtsson C, Phung DC, et al. Seroprevalence of *Helicobacter pylori* infection in urban and rural Vietnam. *Clin Diagn Lab Immunol* 2005;12:81-85 doi: 10.1128/CDLI.12.1.81-85.2005.

For peer review only - http://bmjopen.bmj.com/site/about/guidelines.xhtml

40. Xie C, Lu NH. Review: clinical management of *Helicobacter pylori* infection in China. *Helicobacter* 2015;20:1-10 doi: 10.1111/hel.12178.

41. Yim JY, Kim N, Choi SH, et al. Seroprevalence of *Helicobacter pylori* in South Korea. *Helicobacter* 2007;12:333-40 doi: 10.1111/j.1523-5378.2007.00504.x.

42. Lim SH, Kwon JW, Kim N, et al. Prevalence and risk factors of *Helicobacter pylori* infection in Korea: nationwide multicenter study over 13 years. *BMC Gastroenterol* 2013;13:104. doi: 10.1186/1471-230X-13-104.

43. Moujaber T, MacIntyre CR, Backhouse J, et al. The seroepidemiology of *Helicobacter pylori* infection in Australia. *Int J Infect Dis* 2008;12:500-04 doi: 10.1016/j.ijid.2008.01.011.

44. Mitchell H1, Katelaris P. Epidemiology, clinical impacts and current clinical management of *Helicobacter pylori* infection. *Med J Aust* 2016;204:376-80.

45. Sonnenberg A, Lash RH, Genta RM. A national study of *Helicobactor pylori* infection in gastric biopsy specimens. *Gastroenterology* 2010;139:1894-901 doi: 10.1053/j.gastro.2010.08.018.

46. Cardenas VM, Mulla ZD, Ortiz M, et al. Iron deficiency and *Helicobacter pylori* infection in the United States. *Am J Epidemiol* 2006;163:127-34 doi: 10.1093/aje/kwj018

47. Goh KL, Chan WK, Shiota S, et al. Epidemiology of *Helicobacter pylori* infection and public health implications. *Helicobacter* 2011;16:1-9 doi: 10.1111/j.1523-5378.2011.00874.x.

48. Ueda M, Kikuchi S, Kasugai T, et al. *Helicobacter pylori* risk associated with childhood home environment. *Cancer Sc* 2003;94:914-8. doi: 10.1111/j.1349-7006.2003.tb01375.x

For peer review only

49. Konno M, Fujii N, Yokota S, et al. Five-year follow-up study of mother-to-child transmission of *Helicobacter pylori* infection detected by a random amplified polymorphic DNA fingerprinting method. *J Clin Microbiol* 2005;43:2246-50. doi: 10.1128/JCM.43.5.2246-2250.2005

50. Okuda M1, Miyashiro E, Booka M, et al. *Helicobacter pylori* colonization in the first 3 years of life in Japanese children. *Helicobacter* 2007;12:324-7. doi: 10.1111/j.1523-5378.2007.00510.x

51. The committee of the Japanese Society for Helicobacter Research. [Guidelines for diagnosis and treatment of *H. pylori* infection in Japan: 2016 revised edition]. Tokyo: Sentan Igaku-Sha; 2016. Japanese.

52. Murakami K, Sakurai Y, Shiino M, et al. Vonoprazan, a novel potassium-competitive acid blocker, as a component of first-line and second-line triple therapy for *Helicobacter pylori* eradication: a phase III, randomised, double-blind study. *Gut* 2016;65:1439-46 doi: 10.1136/gutjnl-2015-311304.

For peer review only

For peer review only - http://bmjopen.bmj.com/site/about/guidelines.xhtml

Figure legends

Figure 1. The model used to calculate the number of individuals with successful *H. pylori* eradication.

Figure 2. Annual number of individuals who had successful *H. pylori* eradication (both first-line and second-line). *Others: gastric mucosa associated lymphoid tissue lymphoma, idiopathic thrombocytopenic purpura, and post-endoscopic resection of early gastric cancer.

Figure 3. Trends in (a) the percentages of *H. pylori* infection by birth year based on previous studies and (b) the nationwide number of infected individuals as estimated based on the previous studies. *Data from the study by Watabe et al.[31] were excluded in (a) as that study divided the age group into 2 age groups: below and above age 60. *H. pylori, Helicobacter pylori.*

Figure 4. Trend and prediction of the number of *H. pylori* infected individuals. *Case 0, in current policy; †case 1, if policy change had not occurred in 2000; ‡case 2, if policy change had not occurred in 2013. *H. pylori, Helicobacter pylori.*

For peer review only

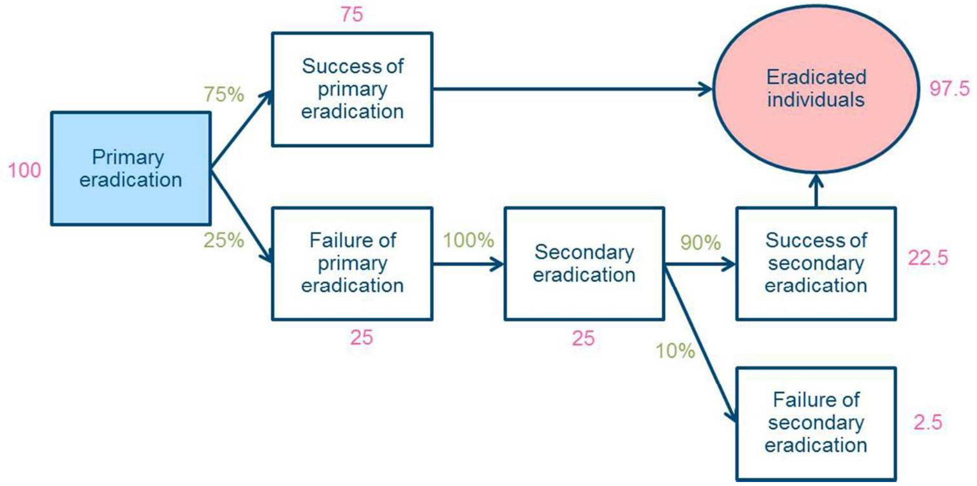

Figure 1. The model used to calculate the number of individuals with successful *H. pylori* eradication.

203x98mm (150 x 150 DPI)

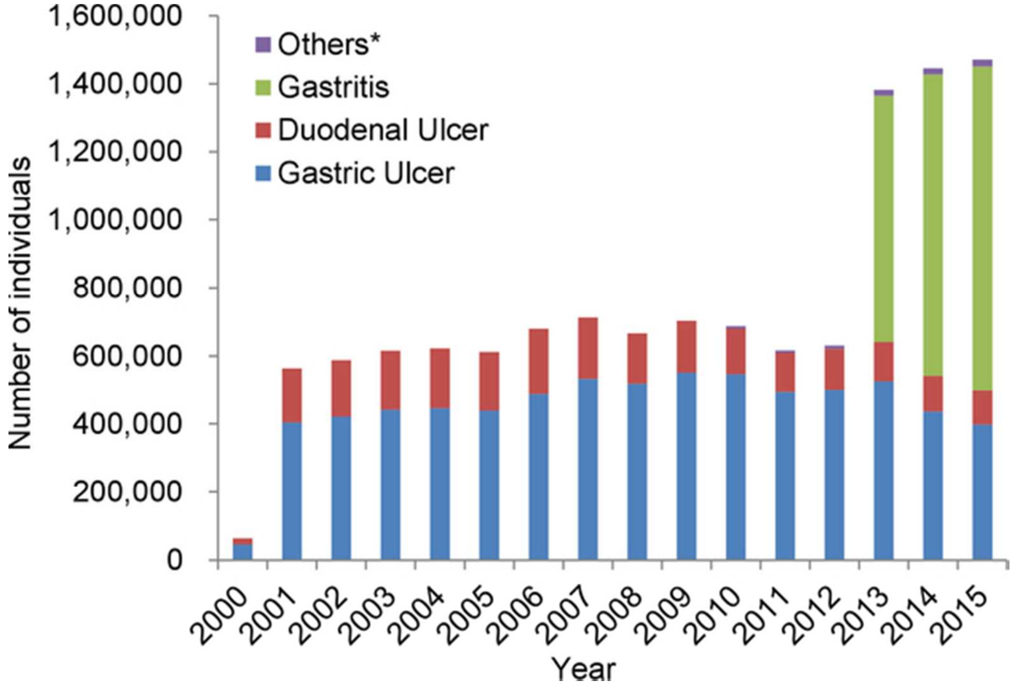

Figure 2. Annual number of individuals who had successful *H. pylori* eradication (both first-line and second-line). *Others: gastric mucosa associated lymphoid tissue lymphoma, idiopathic thrombocytopenic purpura, and post-endoscopic resection of early gastric cancer.

53x35mm (300 x 300 DPI)

For peer review only

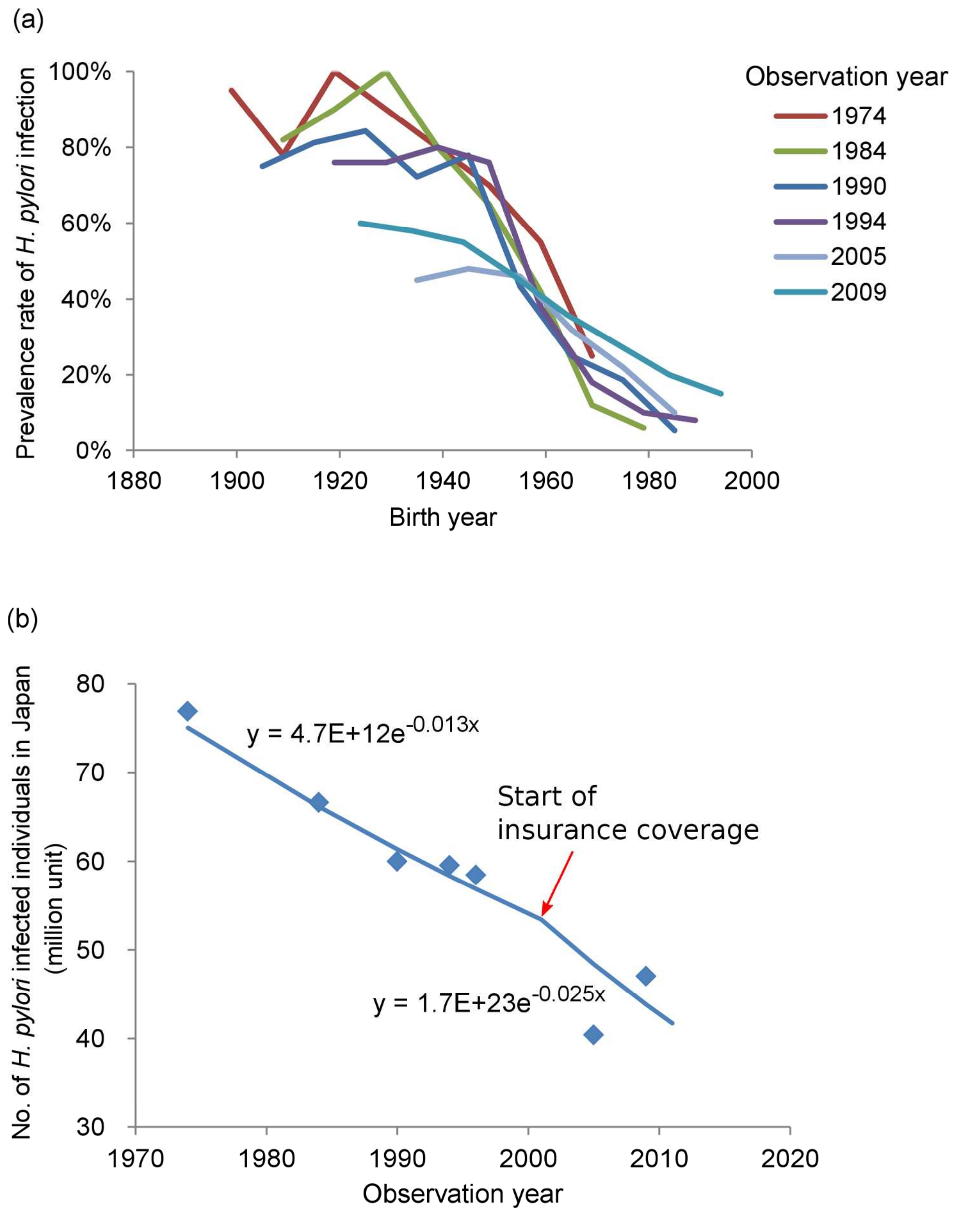

Figure 3. Trends in (a) the percentages of *H. pylori* infection by birth year based on previous studies and (b) the nationwide number of infected individuals as estimated based on the previous studies. *Data from the study by Watabe et al.31 were excluded in (a) as that study divided the age group into 2 age groups: below and above age 60. *H. pylori, Helicobacter pylori*.

746x951mm (72 x 72 DPI)

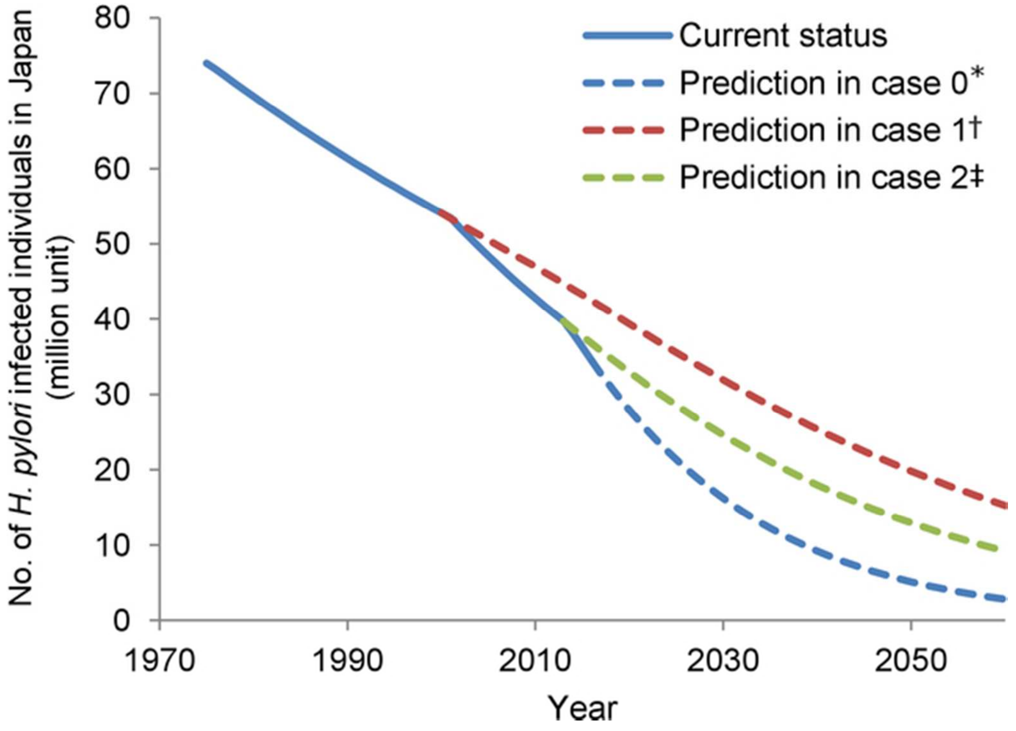

Figure 4. Trend and prediction of the number of *H. pylori* infected individuals. *Case 0, in current policy; †case 1, if policy change had not occurred in 2000; ‡case 2, if policy change had not occurred in 2013. *H. pylori, Helicobacter pylori*.

54x38mm (300 x 300 DPI)

Supplementary appendix

Table S1. The number of primary eradication individuals, mean age, and percentage of males for each year in the JMDC and MDV databases.

		2005	2006	2007	2008	2009	2010	2011	2012	2013	2014	2015
JMDC	Number	1,001	987	1,049	1,426	2,078	3,231	3,985	4,649	19,341	21,951	21,421
	Mean age	41.5	41.1	40.4	39.2	38.4	37.7	38.4	38.3	35.8	37.1	35.9
	(SD)	(9.7)	(9.8)	(9.8)	(10.6)	(10.8)	(10.7)	(10.8)	(10.6)	(10.2)	(10.4)	(10.4)
	Male %	83	79	77	70	67	66	65	65	58	80	58
MDV	Number	-	-	-	442	2,108	5,655	7,520	9,588	36,811	52,920	55,949
	Mean age	-	-	-	58.1	57.9	59.0	59.0	59.3	60.6	60.9	61.0
	(SD)				(14.3)	(13.7)	(14.1)	(14.1)	(13.8)	(12.6)	(12.7)	(12.9)
	Male %	-	-	-	65	66	64	64	64	57	55	55

JMDC, Japan Medical Data Center; MDV, Medical Data Vision; SD, standard deviation.

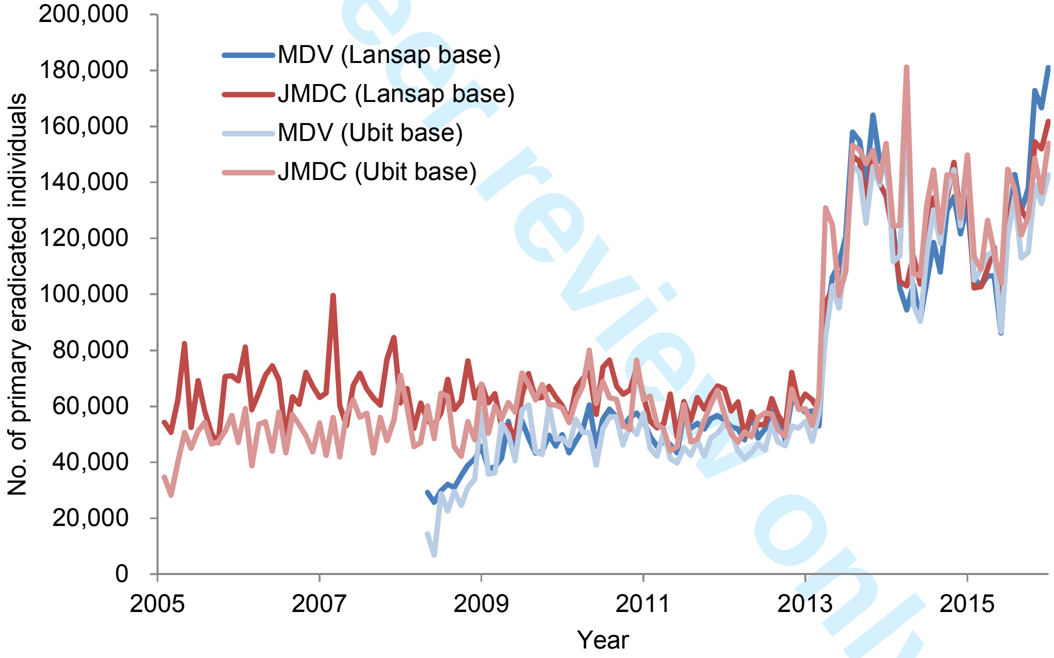

Figure S1. The number of individuals with the primary eradication of *H. pylori* according to each database.

For peer review only

The RECORD statement – checklist of items, extended from the STROBE statement, that should be reported in observational studies using routinely collected health data.

	Item No.	STROBE items	Location in manuscript where items are reported	RECORD items	Location in manuscript where items are reported
**Title and abstract**					
	1	(a) Indicate the study's design with a commonly used term in the title or the abstract (b) Provide in the abstract an informative and balanced summary of what was done and what was found	Title and Abstract; page 1–2.	RECORD 1.1: The type of data used should be specified in the title or abstract. When possible, the name of the databases used should be included.<br><br>RECORD 1.2: If applicable, the geographic region and timeframe within which the study took place should be reported in the title or abstract.<br><br>RECORD 1.3: If linkage between databases was conducted for the study, this should be clearly stated in the title or abstract.	Mentioned the type of data in Title and Abstract; page 1–2.<br><br>Reported the region in Title and Abstract; page 1–2.<br><br>N/A
**Introduction**					
Background rationale	2	Explain the scientific background and rationale for the investigation being reported	The 1st, 2nd and 3rd paragraphs of Introduction; page 5–7.		
Objectives	3	State specific objectives, including any prespecified hypotheses	The 4th paragraph (last paragraph) of Introduction; page 7.		
**Methods**					
Study Design	4	Present key elements of study design early in the paper	Methods (Study design section); page 9.		
Setting	5	Describe the setting, locations, and relevant dates, including periods of recruitment, exposure, follow-up, and data collection	Methods (Data sources section) ; page 7–8.		

For peer review only

Participants	6	*(a) Cohort study* - Give the eligibility criteria, and the sources and methods of selection of participants. Describe methods of follow-up<br>*Case-control study* - Give the eligibility criteria, and the sources and methods of case ascertainment and control selection. Give the rationale for the choice of cases and controls<br>*Cross-sectional study* - Give the eligibility criteria, and the sources and methods of selection of participants<br><br>*(b) Cohort study* - For matched studies, give matching criteria and number of exposed and unexposed<br>*Case-control study* - For matched studies, give matching criteria and the number of controls per case		RECORD 6.1: The methods of study population selection (such as codes or algorithms used to identify subjects) should be listed in detail. If this is not possible, an explanation should be provided.<br><br>RECORD 6.2: Any validation studies of the codes or algorithms used to select the population should be referenced. If validation was conducted for this study and not published elsewhere, detailed methods and results should be provided.<br><br>RECORD 6.3: If the study involved linkage of databases, consider use of a flow diagram or other graphical display to demonstrate the data linkage process, including the number of individuals with linked data at each stage.	The 1st paragraph of Patient identification and statistical analysis section in Methods; page 9–10.<br><br>Provided the detailed methods and results in the 1st paragraph of Patient identification and statistical analysis section in Methods and Patient characteristics section in Results page 9–11, 14.<br><br>N/A
Variables	7	Clearly define all outcomes, exposures, predictors, potential confounders, and effect modifiers. Give diagnostic criteria, if applicable.	Methods (Patient identification and statistical analysis section); page 10–13.	RECORD 7.1: A complete list of codes and algorithms used to classify exposures, outcomes, confounders, and effect modifiers should be provided. If these cannot be reported, an explanation should be provided.	Methods (Patient identification and statistical analysis section); page 10–13.
Data sources/ measurement	8	For each variable of interest, give sources of data and details of methods of assessment	Methods (Patient identification and statistical analysis		

For peer review only

		(measurement). Describe comparability of assessment methods if there is more than one group	section); page 9–11. We used two types of claims databases and two types of product sales data; therefore, four types of combination were obtained and then the combinations were compared (Figure S1)		
Bias	9	Describe any efforts to address potential sources of bias	Discussion (The 2nd paragraph of Strengths and limitations of this study section); page 18–19.		
Study size	10	Explain how the study size was arrived at	Methods (The 1st paragraph of Patient identification and statistical analysis section); page 9–10.		
Quantitative variables	11	Explain how quantitative variables were handled in the analyses. If applicable, describe which groupings were chosen, and why	Methods (Patient identification and statistical analysis section); page 11–13.		
Statistical methods	12	(a) Describe all statistical methods, including those used to control for confounding<br>(b) Describe any methods used to examine subgroups and interactions<br>(c) Explain how missing data were addressed<br>(d) *Cohort study* - If applicable, explain how loss to follow-up was addressed	Methods (Patient identification and statistical analysis section); page 10–13.		

For peer review only

		Case-control study - If applicable, explain how matching of cases and controls was addressed<br>*Cross-sectional study* - If applicable, describe analytical methods taking account of sampling strategy<br>(e) Describe any sensitivity analyses			
Data access and cleaning methods		..		RECORD 12.1: Authors should describe the extent to which the investigators had access to the database population used to create the study population.<br><br>RECORD 12.2: Authors should provide information on the data cleaning methods used in the study.	Methods (Data sources section); page 7.<br><br>N/A
Linkage		..		RECORD 12.3: State whether the study included person-level, institutional-level, or other data linkage across two or more databases. The methods of linkage and methods of linkage quality evaluation should be provided.	N/A
**Results**					
Participants	13	(a) Report the numbers of individuals at each stage of the study (*e.g.*, numbers potentially eligible, examined for eligibility, confirmed eligible, included in the study, completing follow-up, and analysed)<br>(b) Give reasons for non-participation at each stage.<br>(c) Consider use of a flow diagram	Results (Patient characteristics section); page 14.	RECORD 13.1: Describe in detail the selection of the persons included in the study (*i.e.,* study population selection) including filtering based on data quality, data availability and linkage. The selection of included persons can be described in the text and/or by means of the study flow diagram.	Results (Patient characteristics section); page 14.
Descriptive data	14	(a) Give characteristics of study	Results (Patient		

For peer review only - http://bmjopen.bmj.com/site/about/guidelines.xhtml

		participants (*e.g.*, demographic, clinical, social) and information on exposures and potential confounders<br>(b) Indicate the number of participants with missing data for each variable of interest<br>(c) *Cohort study* - summarise follow-up time (*e.g.*, average and total amount)	characteristics section and Table S1); page 14.		
Outcome data	15	*Cohort study* - Report numbers of outcome events or summary measures over time<br>*Case-control study* - Report numbers in each exposure category, or summary measures of exposure<br>*Cross-sectional study* - Report numbers of outcome events or summary measures	Results (Trend in the number of individuals with the primary eradication therapy and successful eradication section); page 14.		
Main results	16	(a) Give unadjusted estimates and, if applicable, confounder-adjusted estimates and their precision (e.g., 95% confidence interval). Make clear which confounders were adjusted for and why they were included<br>(b) Report category boundaries when continuous variables were categorized<br>(c) If relevant, consider translating estimates of relative risk into absolute risk for a meaningful time period	Results (Trend in the number of individuals with the primary eradication therapy and successful eradication section and Figure 1, and Trend and prediction of H. pylori infection and the effect of insurance policy changes section and Figure 3); page 14–15.		
Other analyses	17	Report other analyses done—e.g., analyses of subgroups and interactions, and sensitivity	N/A		

For peer review only

		analyses			
**Discussion**					
Key results	18	Summarise key results with reference to study objectives	The 1st paragraph of Discussion; page 15–16.		
Limitations	19	Discuss limitations of the study, taking into account sources of potential bias or imprecision. Discuss both direction and magnitude of any potential bias	Discussion (The 2nd paragraph of Strengths and limitations of this study section); page 18-19.	RECORD 19.1: Discuss the implications of using data that were not created or collected to answer the specific research question(s). Include discussion of misclassification bias, unmeasured confounding, missing data, and changing eligibility over time, as they pertain to the study being reported.	Discussion (The 2nd paragraph of Strengths and limitations of this study section); page 18-19.
Interpretation	20	Give a cautious overall interpretation of results considering objectives, limitations, multiplicity of analyses, results from similar studies, and other relevant evidence	The 2nd, 3rd and 4th paragraphs of Discussion; page 16-17.		
Generalisability	21	Discuss the generalisability (external validity) of the study results	Discussion (The 2nd and 3rd paragraphs of Strengths and limitations of this study section) ;page 18-19.		
**Other Information**					
Funding	22	Give the source of funding and the role of the funders for the present study and, if applicable, for the original study on which the present article is based	Footnote (Funding); page 20.		
Accessibility of protocol, raw data, and programming code				RECORD 22.1: Authors should provide information on how to access any supplemental information such as the study protocol, raw data, or programming code.	N/A

For peer review only

*Reference: Benchimol EI, Smeeth L, Guttmann A, Harron K, Moher D, Petersen I, Sørensen HT, von Elm E, Langan SM, the RECORD Working Committee. The REporting of studies Conducted using Observational Routinely-collected health Data (RECORD) Statement. *PLoS Medicine* 2015; in press.

*Checklist is protected under Creative Commons Attribution (CC BY) license.

For peer review only
